# Soft Polyethylene Glycol Hydrogels Support Human PSC Pluripotency and Morphogenesis

**DOI:** 10.1021/acsbiomaterials.4c00923

**Published:** 2024-06-19

**Authors:** Michael P. Seitz, Yuanhui Song, Xiaojun Lance Lian, Zhen Ma, Era Jain

**Affiliations:** † Department of Biomedical and Chemical Engineering, Syracuse University, Syracuse, New York 13244, United States; ‡ Bioinspired Syracuse: Institute for Material and Living Systems, Syracuse University, Syracuse, New York 13244, United States; § Department of Biomedical Engineering, The Huck Institutes of the Life Sciences, Department of Biology, Pennsylvania State University, University Park, Pennsylvania 16802, United States

**Keywords:** lumenogenesis, morphogenesis, epiblast, hPSC, PEG hydrogel, mechanobiology

## Abstract

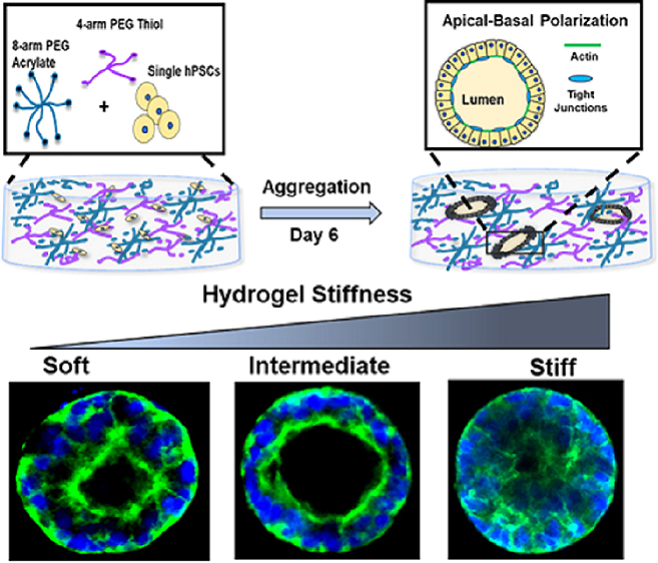

Lumenogenesis within the epiblast represents a critical step in early human development, priming the embryo for future specification and patterning events. However, little is known about the specific mechanisms that drive this process due to the inability to study the early embryo in vivo. While human pluripotent stem cell (hPSC)-based models recapitulate many aspects of the human epiblast, most approaches for generating these 3D structures rely on ill-defined, reconstituted basement membrane matrices. Here, we designed synthetic, nonadhesive polyethylene glycol (PEG) hydrogel matrices to better understand the role of matrix mechanical cues in iPSC morphogenesis, specifically elastic modulus. First, we identified a narrow range of hydrogel moduli that were conducive to the hPSC viability, pluripotency, and differentiation. We then used this platform to investigate the effects of the hydrogel modulus on lumenogenesis, finding that matrices of intermediate stiffness yielded the most epiblast-like aggregates. Conversely, stiffer matrices impeded lumen formation and apico-basal polarization, while the softest matrices yielded polarized but aberrant structures. Our approach offers a simple, modular platform for modeling the human epiblast and investigating the role of matrix cues in its morphogenesis.

## 1 Introduction

Lumenogenesis is a crucial yet poorly understood process responsible for the formation of key morphological features in several tissues and organs.^1^ One of the first and foremost lumenogenesis events occurs during early embryogenesis within the epiblast, a spherical cluster of cells which serve as the precursors to all bodily tissues and organs.^2^ Epiblast lumenogenesis is critical to human development, forming the basis for the amniotic cavity and the spatial organization required for gastrulation.^2,3^ Incorrect formation of this fundamental lumen cavity is known to lead to defects and embryonic lethality.^1,3,4^ Despite the importance of this process, it remains understudied due to our inability to observe the developing human embryo in vivo, especially during early stages of implantation.^5,6^

Cultures of human embryo beyond peri-implantation stages have shown that epiblast lumenogenesis can occur in the absence of maternal tissue, indicating the intrinsic self-organization capability of early embryonic cells.^7,8^ Lumenogenesis as a characteristic behavior of primed human pluripotent stem cells (hPSCs) was first established in a seminal study by Taniguchi et al. in 2015, in which hPSCs were three-dimensionally (3D) cultured in Matrigel, a basement membrane (BM) derived extracellular matrix (ECM), under self-renewal conditions. The resulting cysts were found to be structures with apical-basal polarity and had the innate ability to form a central lumen.^9,10^ Due to their molecular and structural similarities to the developing epiblast, 3D cultured hPSCs provide a model for investigating the mechanisms behind lumenogenesis during early embryogenesis.^5,11−16^

Multiple in vitro hPSC-based embryo models reinforce the fact that there is a tight coupling of chemical and mechanical cues to guide stem cell morphogenesis and create a distinct tissue pattern during early embryogenesis.^5^ Moreover, recent studies show that primed hPSCs are mechanically responsive and maintain self-renewal capacity on surfaces that match their intrinsic softness.^17,18^ Despite this, most work in the field has focused on the molecular mechanisms behind embryonic lumenogenesis, leaving the impact of matrix mechanical cues on this process relatively unexplored. Additionally, established methods for modeling the human epiblast traditionally rely on Matrigel, which presents a barrier due to its ill-defined ECM composition.^5,14,19,20^

While Matrigel provides a 3D microenvironment with the biochemical properties necessary to support hPSC growth, the biochemical and mechanical properties of these matrices are difficult to control, making them unsuitable for investigating the role of ECM mechanical properties in epiblast lumenogenesis.^21−23^ A recent study by Schindler et al. used nonadhesive agarose gels along with growth factor reduced Matrigel to study lumenogenesis in primed hPSCs. The encapsulated hPSCs self-organized into polarized epiblast spheroids under both self-renewing and differentiating conditions.^24^ However, this study did not investigate the impact of the matrix mechanical properties on hPSC lumenogenesis. Another study by Indana et al. studied lumenogenesis in human induced pluripotent stem cell (hiPSC) aggregates formed in alginate hydrogels, finding the adhesion ligand arginine–glycine–aspartate (RGD) and faster stress relaxation to be critical for increasing cell viability and incidence of lumen formation.^25,26^ These two studies clearly indicate the importance of engineered hydrogels in elucidating the role of specific ECM cues in regulating hPSC lumenogenesis, while a deficiency of similar such studies points to a need for a robust platform allowing independent modulation of the biophysical properties such as degradation, mechanical strength, and presentation of bioactive factors to understand the effect of substrate/ECM mechanical properties on epiblast morphogenesis.^21−23,27^

Polyethylene glycol (PEG)-based synthetic hydrogels provide a tunable 3D substrate with physical properties similar to those of natural tissues. PEG hydrogels also enable the generation of modular components to create customized microenvironments by independently tuning hydrogel degradation, stiffness, and tethered or soluble ECM proteins.^28^ Studies using PEG-based synthetic hydrogels have also been used to generate luminal organoids from several cell types, including Madin–Darby canine kidney (MDCK) cells, intestinal stem cells (ISCs), and hiPSCs.^28−30^ However, to the best of our knowledge, a mechanobiological understanding of hPSC morphogenesis and epiblast lumenogenesis using a completely synthetic platform, such as PEG hydrogels, has not yet been achieved.

In this study, we utilized Michael-addition cross-linking to fabricate a set of completely synthetic PEG hydrogels with tunable elastic moduli. We show that upon 3D encapsulation in these hydrogels, hPSCs maintain their pluripotency and form uniformly sized aggregates with a lumen. Like the human epiblast, these aggregates readily self-organize into a radial structure, possess apical-basal polarity, and can freely undergo trilinear differentiation upon stimulation. Strikingly, apical-basal polarization, radial organization, and lumen morphology were found to be highly dependent upon the mechanical stiffness of the hydrogel. Cells grown in substrates of intermediate stiffness assembled into more physiologically relevant, polarized aggregates with a central lumen; stiffer substrates led to no polarization or lumen formation, and soft substrates led to the formation of polarized structures, which tended to have multiple cell layers and asymmetrically placed lumens. Thus, this study establishes a novel, completely synthetic hydrogel platform to study epiblast lumenogenesis and reveals the role of matrix mechanical stiffness in hPSC morphogenesis.

## 2 Materials and Methods

### 2.1 Hydrogel Fabrication and Mechanical Characterization

Stock solutions (20% w/v) of 8-arm PEG acrylate 20 kDa (Jenkem Technology USA, Plano, Texas, USA) and 4-arm PEG thiol 10 kDa (Laysan Bio, Arab, Alabama, USA) were first prepared in 100 mM HEPES buffer (pH 8). The two macromers were mixed in an equimolar ratio of acrylate to thiol groups and diluted to the desired polymer concentration. Aliquots of hydrogel precursor solution were then sandwiched between two parafilm coated glass slides separated by 600 μm silicone spacers and incubated at 37 °C for 1.5 h. After the gelation was completed, the disc-shaped gels were removed from the slides and transferred to 1× PBS (pH 7.4) for washing. In all experiments, samples were allowed to incubate for at least 24 h post-fabrication in 1× PBS at 37 °C. For every experiment, at least three gels were fabricated per condition tested.

Rheological characterization of hydrogels was conducted on a DR-H3 rheometer using an 8 mm plate (TA Instruments, New Castle, Delaware, USA). To determine initial storage (*G*′) and loss (*G*′′) modulus, 50 μL gels were allowed to swell for 24 h post-fabrication at 37 °C. Prior to use, gels were cut into 8 mm discs using a biopsy punch. A frequency sweep was then conducted at 1–10 rad/s under 1% strain, which was determined to be within the linear viscoelastic range. To measure changes in modulus over time, we periodically analyzed hydrogels over the period of time indicated. Young’s modulus was estimated using the formula *E* = 2*G*′(1 + *v*), where Poisson’s ratio (*v*) was held to be 0.5.^31^

### 2.2 Cell Maintenance

WTC-11 (Bruce Conklin’s lab, Gladstone Institutes) and RUES2-GLR (Dr. Ali Brivanlou’s lab at The Rockefeller University) cells were maintained in 2D on Matrigel (Fisher Scientific, Agawam, Massachusetts, USA) coated tissue culture plates in Essential-8 (E8) medium (Fisher Scientific) at 37 °C in 5% CO_2_. The medium was changed daily, and cells were passaged at ∼70% confluency with Accutase (Fisher Scientific). ROCK inhibitor Y-27632 (Stemcell Technologies, Vancouver, British Columbia, Canada) was used at a concentration of 10 μM for the first 24 h post-dissociation to improve cell viability.

### 2.3 Encapsulating Cells within PEG Hydrogels

To encapsulate cells, hydrogels were fabricated as described previously. Macromers were first weighed into microcentrifuge tubes and UV sterilized in a biosafety cabinet for 15 min, after which they were reconstituted into 20% w/v stock solutions with sterile HEPES buffer. Cells were then dissociated with Accutase to form a single cell suspension, which was then mixed with buffer and macromer stocks to achieve the desired cell density and polymer concentration. To prevent dissociation-induced apoptosis, Y-27632 was added to the gel precursor solution at a 10 μM concentration. To prevent cells from settling to the bottom of the gel before gelation, precursor solutions for 1.5% and 2% hydrogels were allowed to pre-react in a cell culture incubator at 37 °C for 7 min to increase the precursor solutions’ viscosity, after which cells were resuspended by pipetting. As the gelation time of 3% gels was considerably faster, this precursor solution was used directly. After hydrogel precursors were prepared, 20 μL aliquots were placed between presterilized parafilm coated glass slides with 600 μm silicone spacers. The slides containing hydrogel aliquots were then placed in a cell incubator maintained at 37 °C and 5% CO_2_, where they were left to gel for 1.5 h. After gelation was complete, hydrogels with encapsulated cells were transferred to tissue culture treated 48-well plates. Each sample was incubated in 300 μL of E8 medium supplemented with 10 μM Y-27632. The medium in all samples was refreshed daily. All samples were maintained for 3 or 6 days, as needed.

### 2.4 Viability Staining

hiPSCs encapsulated in hydrogels were stained using the LIVE/DEAD Viability/Cytotoxicity Kit (Fisher Scientific), which was used as per the manufacturer’s protocol. Briefly, hydrogel samples were washed with 1× PBS once before incubation with a calcein AM/ethidium homodimer working solution for 30 min at 37 °C in 5% CO_2_. After incubation, samples were washed once and imaged on a Leica Thunder DM4 B microscope (Leica Microsystems, Deerfield, Illinois, USA) using a 10× objective. At least three images were taken per sample.

### 2.5 Immunostaining

At the specified time points, gels were fixed with 4% paraformaldehyde (PFA) at room temperature (RT) for 15 min. All gels were washed three times in PBS for 5 min, and simultaneously blocked and permeabilized with 1% BSA and 0.3% triton X-100 for 1 h at RT. After the gels were washed three times for 5 min each with PBS, they were incubated with primary antibody (Table S1) in PBS with tween (PBST) overnight at 4 °C. Appropriate isotype controls were included in each experiment. After three 45 min washes at RT with PBS, gels were incubated with the appropriate secondary antibody in PBST overnight at 4 °C. After three 45 min washes with PBS, samples were counterstained with DAPI for 1 h. Gels were washed for three times, 10 min each, in PBS and imaged. All images were taken on either a Zeiss LSM 710 or 980 confocal microscope. At least three samples were analyzed per marker, with at least three images taken per sample to cover the entire gel area. All antibodies used in the study are listed in Table S1.

### 2.6 Actin Staining and Lumen Imaging

Aggregate F-actin was stained with Phalloidin-iFluor 488 (Abcam), according to the manufacturer’s protocol, and counterstained with DAPI for 1 h. Imaging was performed on a Zeiss LSM 710 confocal microscope with at least three images taken per sample. To more precisely capture lumens, the *z*-stack images with a approximate step size of 5 μm were taken (Supplementary Video 1).

### 2.7 Trilineage Differentiation

Before stimulating differentiation, encapsulated hPSCs were grown for 4 days under self-renewing conditions. To induce endoderm and ectoderm differentiation, E8 was switched to Stemdiff Trilineage Endoderm and Ectoderm media (Stemcell Technologies) supplemented with 10 μM Y-27632 for 4 and 6 days, respectively. Mesoderm induction was adapted from Arkenberg et al., in which E8 was switched to RPMI 1640 containing B27 supplement minus insulin, 6 μM CHIR-99021 (Stemcell Technologies), and 10 μM Y-27632 for 2 days.^32^ After differentiation, samples were prepared for immunostaining and imaging, as described earlier.

### 2.8 Image Analysis

ImageJ was used for the image analysis for all experiments.

#### 2.8.1 Viability and Aggregate Morphology

Samples stained for viability were used to quantify aggregate viability, diameter, and circularity. Viability was determined by separately thresholding the live and dead channels and using the analyze particles function to measure the total fluorescent area. Viability was then expressed as the ratio of the total viable cell area against the total dead area. Aggregate circularity was next measured according to the method described by Indana et al.^25^ Using the same live channel mask, the watershed, fill holes, and median functions (2.0 pixels) were used before the analyze particles function with “Shape Descriptors”. Diameter was then measured by manually drawing a line perpendicularly across the aggregate and using the measure function. As some aggregates had a more elliptical shape, the diameter was measured across the minor axis for every aggregate to keep results consistent.

#### 2.8.2 Cell Alignment and Aspect Ratio

Cell radial alignment and aspect ratio were quantified from E-cadherin immunofluorescence images. A mask was first produced using the threshold function in ImageJ, after which the lookup table (LUT) was inverted. The median filter was then applied at 2.0 pixels, followed by fill holes and watershed segmentation. The aspect ratio, Feret angle, and centroid were found using the analyze particles function. A line was then drawn between the centroid of the cell and the centroid of the lumen or, in the case of aggregates without a lumen, the centroid of the total aggregate (Figure S5). The Feret angle of this line was then measured, and the difference between this angle and the cell Feret angle was reported as the cell radial offset.

#### 2.8.3 Pluripotency, Proliferation, and Trilineage Differentiation

The percentage of cells expressing pluripotency (OCT4, SOX2, and NANOG), proliferation (Ki67), and germ layer markers (PAX6, T-BRA, and SOX17) was found by manually counting the total number of cells per aggregate and the number of cells staining positive for their respective markers.

#### 2.8.4 Lumen Morphology

Volumes were calculated by first acquiring *z*-stacks of phalloidin/DAPI stained aggregates on a Zeiss 710 confocal microscope using a 20× objective. For all stacks, the step size was ∼5 μm. After image acquisition, the phalloidin (green) and DAPI (blue) channels were merged in ImageJ to create a single, grayscale, 8-bit stack using the image calculator plug-in. To calculate the lumen volume, single aggregate stacks were thresholded and analyzed using the volume calculator plug-in. Using the same mask, the total aggregate volume was found after using the fill hole function. Normalized lumen centroid distance was found by first isolating a single *z*-slice from each aggregate, taken from where the lumen reached its maximum diameter. This slice was then a threshold, and the resulting mask was duplicated. To find the centroid of the total aggregate, the fill hole function was used on one of the masks, and the analyze particles function was used to find the centroid coordinates. To find the lumen centroid, a mask of the lumen area was created by inverting the LUT of one of the original masks before the analyze particles function was used. The distance between the total aggregate and lumen centroid coordinates was then calculated. This value was then normalized to the aggregate diameter, which was found by manually using the measure function. To calculate the number of cells per aggregate, nuclei were manually counted by using maximum projections. For all images, the aggregates analyzed were not in contact with any other aggregates.

#### 2.8.5 Actin and ZO-1 Localization

To find the F-actin and ZO-1 intensity profiles across aggregates, a line was first drawn perpendicularly through the center of the lumen, across the entire length of the aggregate. The plot profile function was then used to generate a fluorescence intensity histogram at each point across this line. To compare the profile of different aggregates, the fluorescent intensity and position along the line were normalized to the maximum intensity and position values, yielding values from 0.0 to 1.0. For the position, this corresponded to the left (0) and right (1.0) ends of the aggregate. For fluorescent intensity, the point along the aggregate profile with the greatest fluorescent signal had an intensity of 1.0. The apical and basal intensities of F-actin and ZO-1 were found by manually tracing the apical and basal membranes of each aggregate and measuring the mean fluorescent intensity (MFI).

#### 2.8.6 Statistical Analysis

All calculations and statistical analyses were performed using Graphpad Prism. Unpaired *t* tests and one-way ANOVA with Tukey’s post hoc HSD test were used where applicable. All sample sizes, *p* values, and statistical tests conducted are stated in the legends of each figure.

## 3 Results

### 3.1 PEG Hydrogels Cross-Linked via Michael-Addition Reaction Permit Precise Control over Elastic Modulus

**Figure 1:**
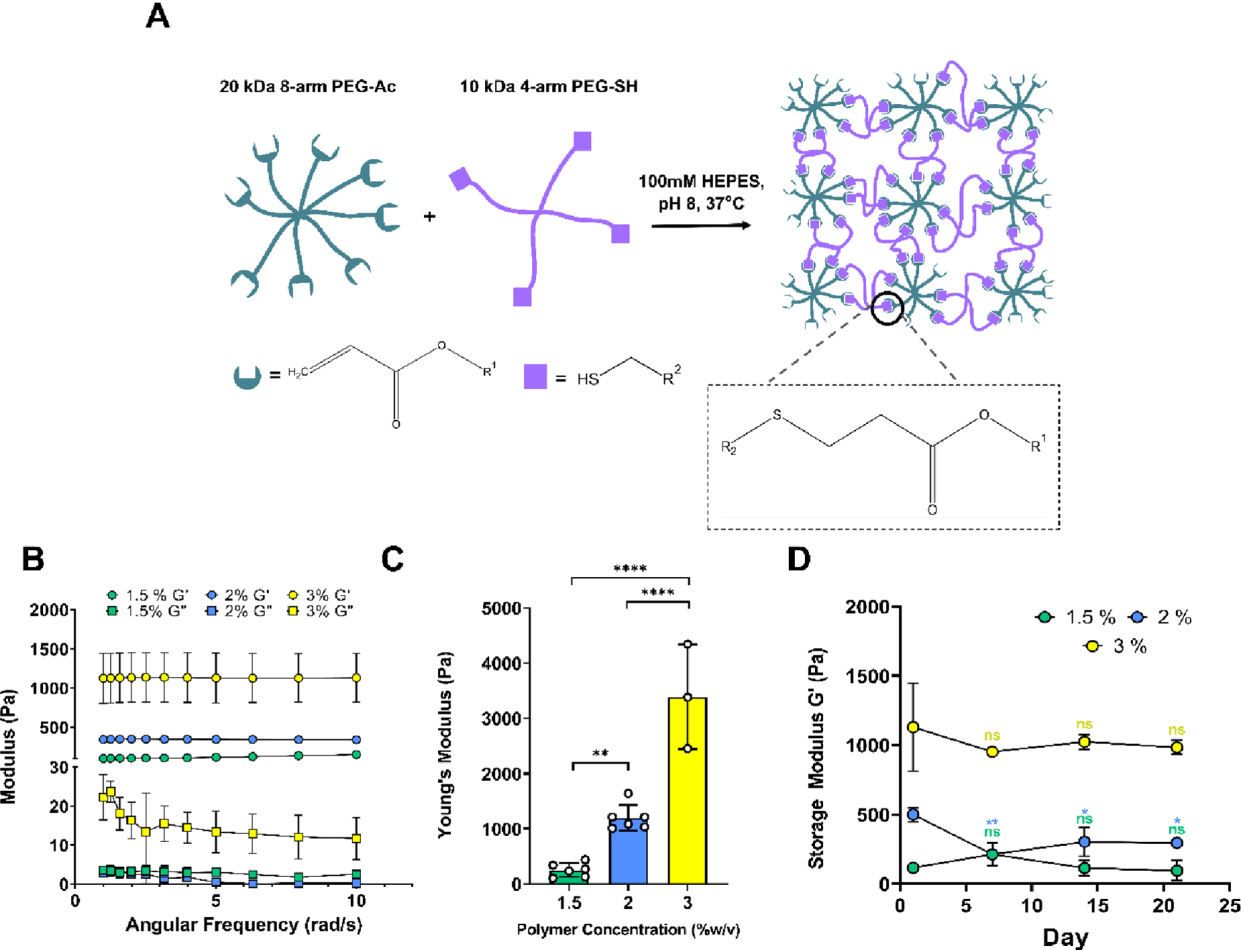
Hydrogel polymer concentration can be used to control the matrix elastic modulus. (A) Schematic depicting the PEG hydrogel fabrication method and reaction chemistry. Multi-arm PEG acrylate and thiol macromers were reacted under mild reaction conditions to form hydrogel matrices. (B) Representative frequency sweep profile of 1.5–3% hydrogels at 1% strain. *G*′ and *G*″ represent storage and loss modulus, respectively. (C) Young’s modulus of hydrogels made using different polymer concentrations as indicated. Young’s modulus was calculated from storage modulus values, as indicated in the Materials and Methods. (D) Changes in hydrogel storage modulus (*G*′) over time. All gels were incubated in PBS buffer at 37 °C until used for measurement. Significance for each time point was relative to the elastic modulus on day 1. Each bar or point represents mean values ± standard deviation (*n* ≥ 3 hydrogels). Ordinary one-way ANOVA with Tukey’s post hoc correction was used to analyze data in each experiment (ns = not significant; *p* > 0.05, *p < 0.05, **p < 0.01, and ****p < 0.0001).

We fabricated the PEG hydrogels using the Michael-addition reaction between the thiol and acrylate to obtain hydrogels with tunable mechanical stiffness. We chose this reaction because of its high specificity, mild gelation conditions, and negligible impact on the cell viability during encapsulations.^27,28^ Our initial studies focused on the screening of different gel forming PEG macromers and their impact on the hydrogel elastic modulus and hiPSC viability (Table S2 and Figures S1and S2). Our results demonstrated that the hydrogel made by combining the 8-arm PEG acrylate and 4-arm PEG thiol macromers showed the highest cell viability and formation of hydrogels at low polymer concentrations. This was an important criterion, as previous studies have shown that the high molecular weight precursors can be used to form very soft PEG hydrogels at low polymer concentration with high reaction efficiency and stable modulus.^33^ Moreover, many studies in the literature indicate that hydrogels with low elastic moduli support high viability in hiPSCs.^22,26,30^ Thus, further hydrogels were made by combining 8-arm PEG acrylate and 4-arm PEG thiol macromers. Hydrogels were made at four different concentrations (1.5–5% w/v) to generate hydrogels with tunable elastic moduli (Figures 1 and S2). Cells were then encapsulated at a density of 5 × 10^6^ cells/mL and maintained for 3 days in E8 medium supplemented with 10 μM Y-27632. While cells remained viable in 1.5–3% w/v hydrogels, 5% w/v hydrogels did not support high cell viability (Figure S2). As such, the 5% w/v hydrogel formulation was excluded from further experiments.

Further, rheological analysis of the remaining formulations demonstrated that the minor changes in polymer concentration had a significant effect on hydrogel stiffness, allowing us to fabricate matrices with Young’s modulus ranging from ∼200 to 3000 Pa (Figure 1C). The storage modulus (*G*′) of all the hydrogels was 10 times the loss modulus and did not change during a frequency sweep measurement, confirming that these hydrogels were primarily elastic (Figure 1B). To determine the hydrolytic stability of each of these hydrogels, the change in modulus was analyzed over time. While modulus did not significantly differ between days 1 and 21 postfabrication for 1.5% and 3% hydrogels, a small but significant decrease was found in 2% gels (Figure 1D). Thus, the use of these PEG macromers allowed us to fabricate highly soft hydrogels at low polymer concentrations. We further designate these three gel formulations as low (1.5% w/v), intermediate (2% w/v), and high stiffness (3% w/v) gels.

### 3.2 Hydrogel Stiffness Affects hiPSC Viability and Aggregate Morphology

We next investigated the effects of hydrogel stiffness on the hiPSC viability and aggregate morphology over a period of 6 days. We first assessed the effect of rho-associated protein kinase inhibition (ROCKi; Y-27632) on cell viability, as its role in preventing dissociation-induced apoptosis in hiPSCs is well established. Cells were encapsulated in intermediate stiffness gels at a density of 2.5 × 10^6^ cells/mL and allowed to aggregate for 3 days in self-renewing conditions, before culturing for a subsequent 3 days without ROCKi. Viability staining revealed a marked decrease in the live cell area when grown without ROCKi, with clusters of dead cells appearing on the exterior of several aggregates (Figure S3). Thus, ROCK inhibition was deemed necessary, and ROCKi was added to all culture conditions for the entire period. Our observations corroborate earlier findings whereby inclusion ROCKi was found necessary for the entire culture period for cell survival upon single hiPSCs culture in synthetic hydrogels.^34^

**Figure 2:**
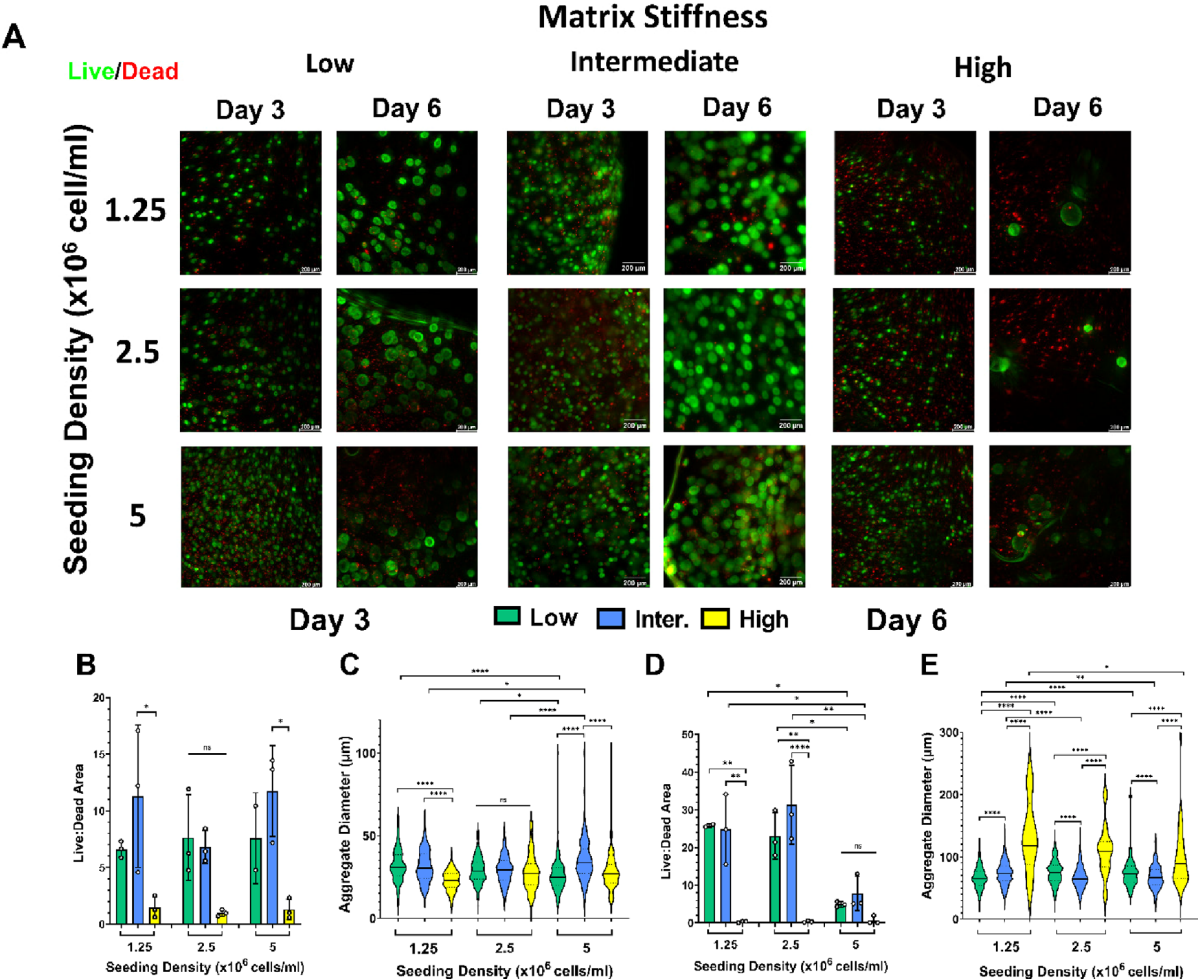
Hydrogel stiffness influences the hiPSC viability and aggregate size. (A) Representative images of live/dead assay performed on cells encapsulated within hydrogels of different stiffness. Cell viability was measured on days 3 and 6 post-encapsulation. Scale bars are 200 μm. (B, D) Quantification of cell viability from images captured during live/dead assay on days 3 and 6 post-encapsulation of single hiPSCs. Viability is represented as the ratio of green to red channel area, averaged across the entire sample (*n* ≥ 3 gels). Cells were encapsulated at three different seeding densities (1.25–5 × 10^6^ cells/mL). (C) Aggregate diameter on day 3 across *n* ≥ 3 gels (1.25–5 × 10^6^ cells/mL; low stiffness gels *n* = 136, 174, 101 aggregates; intermediate stiffness gels *n* = 224, 336, 94 aggregates; and high stiffness gels *n* = 180, 290, 103 aggregates). (E) Aggregate diameter on day 6 across *n* ≥ 3 gels (1.25–5 × 10^6^ cells/mL; low stiffness gels = 313, 435, 5 aggregates; intermediate stiffness gels *n* = 313, 454, 11 aggregates; and high stiffness gels *n* = 205, 274, 39 aggregates). Ordinary one-way ANOVA with Tukey’s post hoc correction was used to analyze each data (ns = not significant; *p* > 0.05, *p < 0.05, **p < 0.01, and ****p < 0.0001).

To model the peri-implantation epiblast, single hiPSCs must be able to proliferate and reorganize to form spherical, multicellular aggregates. To assess the ability of our system to support hiPSC viability and aggregation, we next characterized the effect of seeding density on these properties by encapsulating single hiPSCs at three densities (1.25–5 × 10^6^ cells/mL) in hydrogel formulations with low, intermediate, and high stiffness. Cells were then cultured under self-renewing conditions for 6 days, a time scale that has been found sufficient to assess cell viability and aggregate formation by 3D cultured hiPSCs.^9,35^ In every gel, aggregate formation was observed for 3 days. On days 3 and 6 post-encapsulation, hiPSCs maintained high viability in gels of low and intermediate stiffness for seeding densities of 1.25 and 2.5 × 10^6^ cells/mL. No significant differences in cell viability were found between gels of low and intermediate stiffness at any seeding density, while cell viability was significantly lower in gels of high stiffness (Figure 2A,B,D). On day 6 post-encapsulation, a sharp decrease in viability was observed at the highest seeding density (5 × 10^6^ cells/mL) irrespective of the hydrogel stiffness (Figure 2D). Notably, clusters of dead cells were present in the interior of the gel, while aggregates closer to the outer rim were viable.

Hydrogel stiffness and seeding density also influenced hiPSC aggregate size. On day 3, the aggregate diameter was significantly higher in gels of low and intermediate stiffness compared to gels of high stiffness at low seeding density. However, at the highest seeding density, gels of intermediate stiffness had the largest aggregate diameter. Moreover, increasing the seeding density significantly decreased the aggregate diameter in low stiffness hydrogels, while it increased in hydrogels of intermediate and high stiffness (Figure 2C). These trends were reversed by day 6, and the effect of hydrogel stiffness on aggregate size was more pronounced. On day 6, the aggregate size was largest in the high stiffness gels at all seeding densities, while there were small but significant differences in aggregate size in low and intermediate stiffness gels. Further, by day 6, increasing the seeding density caused a significant decrease in aggregate diameter for gels of intermediate and high stiffness, while aggregates in low stiffness gels increased in size (Figure 2E). Soft and intermediate gels seeded at lower cell densities generally produced the most uniform aggregates (Figure S4). Aggregates were also relatively spherical, with all mean circularities >0.7 (Figure S4). Circularity was not affected by the gel stiffness or seeding density. In summary, these data demonstrate that hydrogel stiffness influences hiPSC viability and aggregate size, whereby there is a narrow range of hydrogel stiffness (200–1200 Pa, soft and intermediate stiffness) that supports hiPSC viability and uniform aggregate formation.

### 3.3 Hydrogel Stiffness Influences Lumenogenesis in hiPSC Aggregates

**Figure 3:**
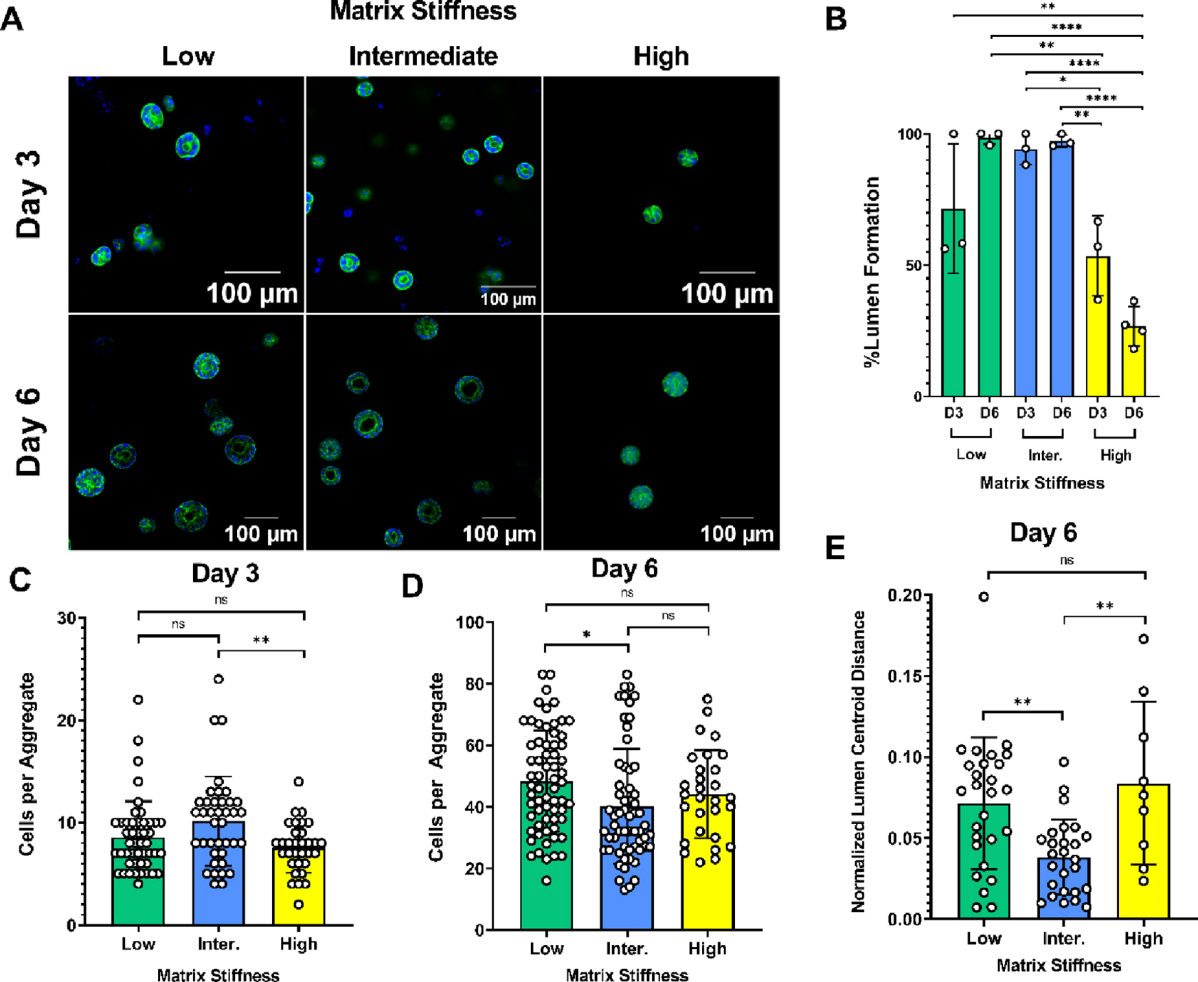
Hydrogel stiffness influences the frequency of lumen formation and the aggregate morphology in hiPSCs. (A) Representative images of F-actin (green) and nucleus (blue) staining through 6 days of culture post-encapsulation of single hiPSCs in hydrogel. Scale bars are 100 μm. (B–E) Morphological characteristics of hiPSCs cultured in different hydrogel matrices, quantified from F-actin staining. (B) Mean percentage of aggregates containing a lumen. The number of aggregates with lumens was first averaged across individual samples before the total mean was calculated on day 3 (*n* ≥ 3 gels, low stiffness gels *n* = 43, intermediate stiffness gels *n* = 45, and high stiffness gels *n* = 39 aggregates) and day 6 (low stiffness gels *n* = 82, intermediate stiffness gels *n* = 219, and high stiffness gels *n* = 49 aggregates). (C, D) Number of cells per aggregate, compared between different hydrogels of varying stiffness on day 3 (low stiffness gels *n* = 47, intermediate stiffness gels *n* = 39, and high stiffness gels *n* = 34 aggregates) and day 6 post-encapsulation in hydrogels (low stiffness gels *n* = 65, intermediate stiffness gels *n* = 60, and high stiffness gels *n* = 29 aggregates). (E) Lumen position, relative to the aggregate, on day 6. Position is represented as the distance between aggregate and lumen centroids, normalized to the diameter of the aggregate (low stiffness gels *n* = 27, intermediate stiffness gels *n* = 26, and high stiffness gels *n* = 9 aggregates). Ordinary one-way ANOVA with Tukey’s post hoc correction was used to compare results between hydrogels of different stiffness (ns = not significant; *p* > 0.05, *p < 0.05, **p < 0.01, and ****p < 0.0001).

Using the library of hydrogel formulations, we identified those conducive to hiPSC culture, we next investigated the impact of hydrogel stiffness on lumenogenesis. We observed the formation of a central lumen in hiPSC aggregates cultured within hydrogels of different stiffness. Actin staining revealed vast differences in aggregate and lumen morphology, depending on the hydrogel stiffness (Figure 3A and Supplementary Video 1). One of the striking impacts of hydrogel stiffness was the incidence of lumen formation by hiPSCs encapsulated in hydrogels. By day 3, ∼70% of aggregates grown in low stiffness (1.5%) gels and ∼90% of those in intermediate stiffness (2%) gels contained lumens, which was significantly greater than those in the high stiffness (3%) gels at ∼55% (Figure 3B).

This gap further widened by day 6, where ∼98% of aggregates in both low and intermediate stiffness gels contained lumens compared to ∼35% in high stiffness gels (Figure 3B). There was a small but significant difference in the number of cells per aggregate between intermediate and high stiffness gels on day 3 and between low and intermediate stiffness gels on day 6 (Figure 3C,D). We also observed that matrix stiffness affected the location at which lumens developed, as aggregates in intermediate stiffness gels had a significantly more centrally placed lumen than aggregates in low and high stiffness gels (Figure 3E). Matrix stiffness also influenced the gross morphology of aggregates. Aggregates organized differently, such as those grown in intermediate stiffness gels, were more likely to form single layers around a central lumen, while aggregates in low and high stiffness gels had multiple (Figure 3A).

**Figure 4:**
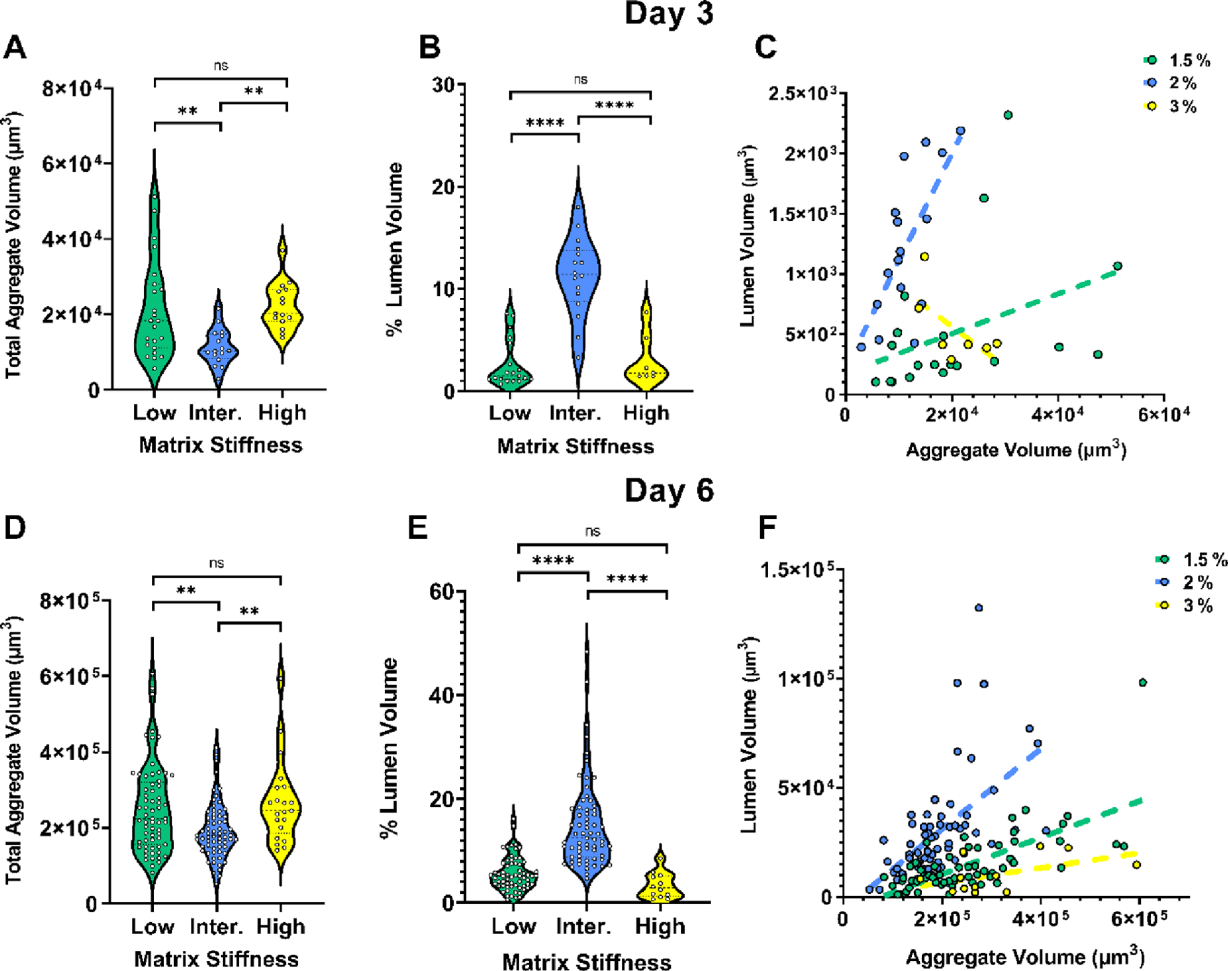
Lumen morphology in hiPSC aggregates is influenced by the hydrogel stiffness. (A–F) Total aggregate volume and the percentage contributed by lumens were quantified from F-actin fluorescent staining images. (A, B) Total aggregate volume and % lumen volume on day 3 (low stiffness gels *n* = 23, intermediate stiffness gels *n* = 16, and high stiffness gels *n* = 15 aggregates). (D, E) Total aggregate volume and % lumen volume on day 6 (low stiffness gels *n* = 64, intermediate stiffness gels *n* = 46, and high stiffness gels *n* = 21 aggregates). Ordinary one-way ANOVA with Tukey’s post hoc correction was used to compare volumes between hydrogels of different stiffness (ns = not significant; *p* > 0.05, **p < 0.01 and ****p < 0.0001). (C, F) Lumen volume was plotted against total aggregate volume for day 3 and day 6 aggregates. A simple linear regression was performed, showing that aggregates clustered based on the hydrogel matrix stiffness they were cultured in.

Lastly, we observed that differences in lumen and total aggregate volume were correlated to the stiffness of the gels in which they were cultured in. Both total aggregate volume and percentage lumen volume increased from day 3 to day 6 (Figure 4A–F). On both days, aggregates grown in gels of intermediate stiffness were found to have a significantly lower total aggregate volume than those grown in the low and high stiffness gels (Figure 4A,B,D,E). Among the aggregates containing a lumen, those grown in intermediate stiffness gels had a significantly larger lumen volume relative to their total aggregate volume, while no difference was found between aggregates in the low and high stiffness gels. When correlating lumen and total aggregate volumes, lumen volume was found to scale more proportionally with total aggregate volume in intermediate stiffness gels. However, in soft and high stiffness gels, as the aggregates grew larger, the lumen volume did not grow correspondingly (Figure 4C,F). This trend was observed on both days 3 and 6. The total aggregate and lumen volumes of aggregates grown in intermediate stiffness gel on day 6 closely agreed with those of day 10 human epiblasts with proamniotic cavity studied in Simunovic et al., based on a meta-analysis of the work presented in Indana et al. (2021).^25,36^

### 3.4 Hydrogel Stiffness Influences Proliferation in hiPSC Aggregates

**Figure 5:**
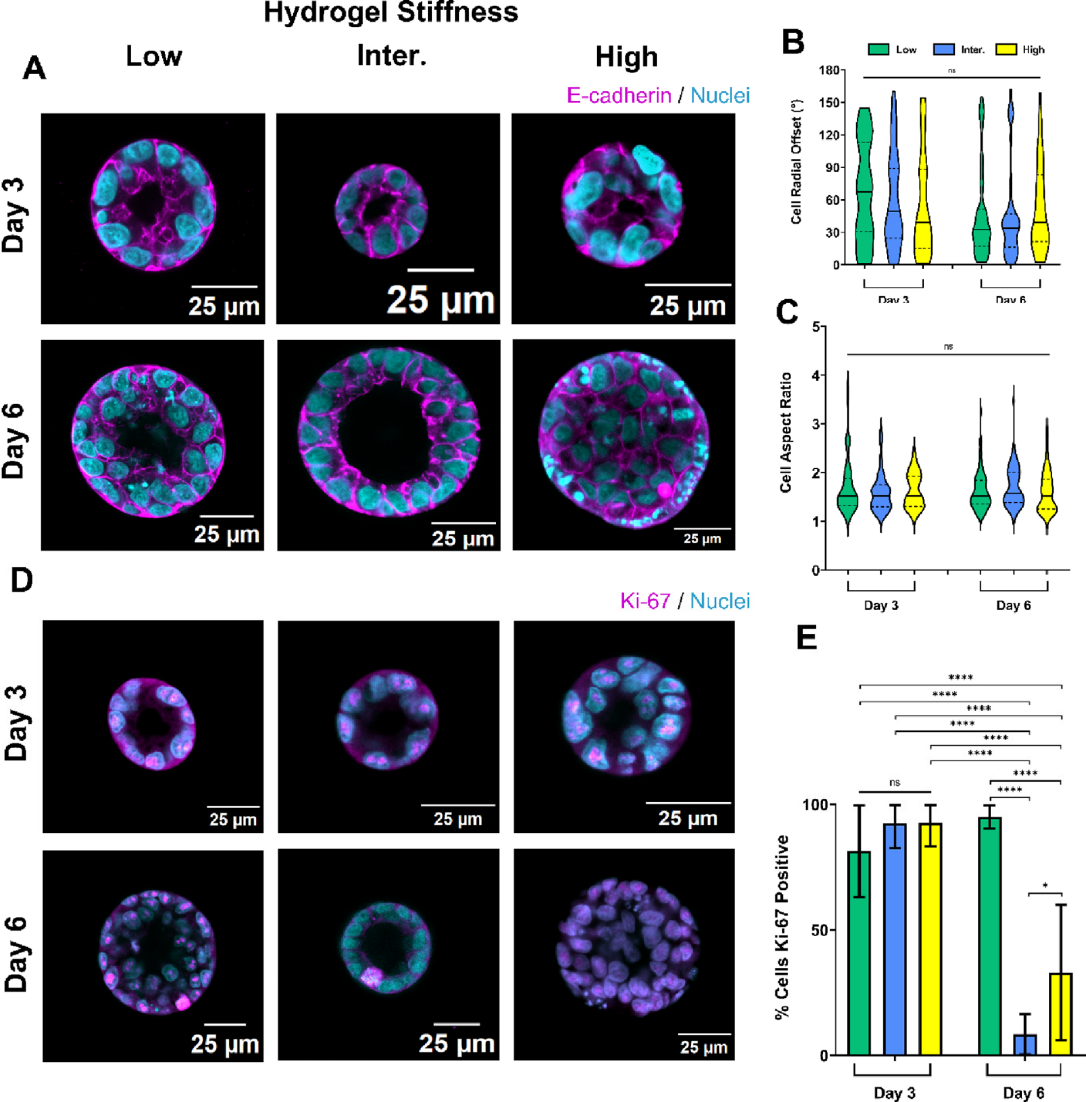
Hydrogel stiffness influences the aggregate organization and proliferation. (A) E-cadherin immunofluorescence of aggregates on days 3 and 6 of culture. Cells were encapsulated at a density of 2.5 × 10^6^ cells/mL. (B, C) Cell radial organization and shape were quantified from E-cadherin immunofluorescent images on day 3 (low stiffness gels *n* = 48, intermediate stiffness gels *n* = 62, and high stiffness gels *n* = 44 cells) and day 6 (low stiffness gels *n* = 85, intermediate stiffness gels *n* = 81, and high stiffness gels *n* = 43 cells). Ordinary one-way ANOVA with Tukey’s post hoc correction was used to compare volumes between hydrogels of different stiffness (ns = not significant). (D) Ki67 immunofluorescence of aggregates on days 3 and 6 of culture. Cells were encapsulated at a density of 2.5 × 10^6^ cells/mL. (E) Percentage of proliferating cells was calculated using the equation (number of Ki67+ cells/total number of cells) × 100, on day 3 (low stiffness gels *n* = 10, intermediate stiffness gels *n* = 9, and high stiffness gels *n* = 5 aggregates) and day 6 (low stiffness gels *n* = 9, intermediate stiffness gels *n* = 12, and high stiffness gels *n* = 4 aggregates). Ordinary one-way ANOVA with Tukey’s post hoc correction was used to compare differences between hydrogels of different stiffness (ns = not significant).

Lumenogenesis is a tightly controlled process, in which the interplay between controlled cell division and cell–cell adhesion has been shown to contribute to cell orientation and apical-basal polarization.^37,38^ To understand the role of these factors in our system, cell adhesion molecule E-cadherin and proliferation marker Ki67 were analyzed through immunofluorescence. At all matrix stiffnesses and on both days 3 and 6 post-encapsulation, E-cadherin was expressed robustly at cell–cell interfaces (Figure 5A). Cell alignment within aggregates grown at all stiffnesses was found to be relatively disorganized at day 3 with an increase in radial orientation toward the lumen in all conditions by day 6, though this was not found to be statistically significant (Figure 5B). No difference in the cellular aspect ratio was observed between days or matrix stiffnesses (Figure 5C). Despite large differences in aggregate viability between different hydrogel stiffnesses, immunofluorescent analysis of Ki67 revealed over 80% of cells in all hydrogels to be proliferating on day 3 (Figure 5D,E), indicating that cells that were able to aggregate by this point maintained their capacity for proliferation. This percentage remained consistent in soft hydrogels by day 6 but exhibited a notable reduction in Ki67 expression in intermediate and stiff hydrogels, indicating a decline in cell proliferation.

### 3.5 Matrix Stiffness Influences Apical-Basal Polarization in Lumen-Containing hiPSC Aggregates

**Figure 6:**
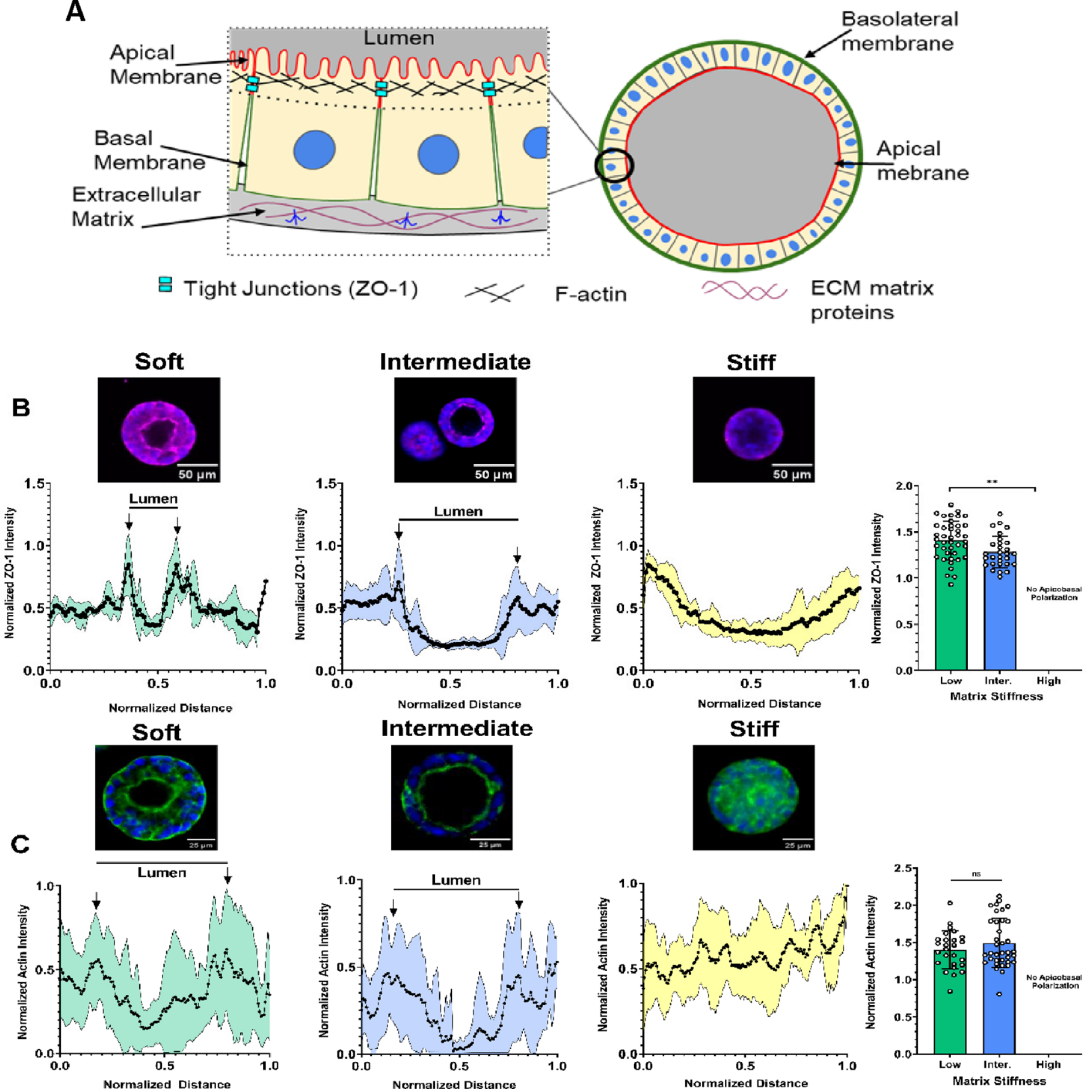
Hydrogel stiffness regulates the apical-basal polarization in hiPSC aggregates. (A) Schematic showing self-organization of hiPSCs around a central lumen and acquiring apical-basal polarity with a difference in distribution of cellular components on the apical and basal side as shown. (B) Apical localization of tight junction protein ZO-1 was quantified from immunofluorescence images. Scale bars on representative images are 50 μm. Mean fluorescent intensity profiles were derived from the histograms of multiple aggregates (low stiffness gels *n* = 11, intermediate stiffness gels *n* = 7, and high stiffness gels *n* = 6 aggregates). Arrows mark fluorescent maxima along the apical membrane. Mean apical ZO-1 intensity between conditions (low stiffness gels *n* = 41, and intermediate stiffness gels *n* = 29 aggregates) was analyzed using an unpaired, two-tailed *t* test with Welch’s correction (**p < 0.01). (C) Apical localization of actin was quantified through phalloidin staining. Scale bars on representative images are 25 μm. Mean fluorescent intensity profiles were derived from the histograms of multiple aggregates (low stiffness gels *n* = 9, intermediate stiffness gels *n* = 10, and high stiffness gels *n* = 8 aggregates). Arrows mark fluorescent maxima along the apical membrane. Mean apical actin intensity between conditions (low stiffness gels *n* = 26 and intermediate stiffness gels *n* = 36 aggregates) was analyzed using an unpaired, two-tailed *t* test with Welch’s correction (**p < 0.01).

One of the hallmarks of the peri-implantation epiblast is the development of apical-basal polarity, in which proteins involved in lumenogenesis such as F-actin or tight junction protein ZO-1 become enriched in the apical membrane, while basement membrane components become enriched on the basolateral membrane^9,39,40^ (Figure 6A). As both actin polymerization and the maturation of tight junctions have been described as key players in the initial formation and expansion of lumens, we chose to analyze how matrix stiffness influenced the cellular localization of F-actin and the tight junction protein ZO-1.^39,40^ Strikingly, almost all lumen-containing aggregates in low and intermediate stiffness gels stained positive for ZO-1 on day 6, while none were positive in the high stiffness gels (Figure 6B). In gels of low and intermediate stiffness, fluorescent intensity profiles across aggregates revealed two distinct peaks corresponding to the apical sides of the lumen, indicating apical localization. Apical localization was not present in aggregates grown in high stiffness gels. When comparing low and intermediate stiffness gels, where polarized lumens formed, aggregates in low stiffness gels were found to have significantly higher apical mean fluorescent intensity (MFI) values relative to the basal MFI, suggesting a more polarized expression. When plotting actin fluorescence in the same manner, we found a similar apical localization on day 6 in aggregates from low and intermediate stiffness gels with most aggregates in high stiffness gels remaining unpolarized (Figure 6B,C). Rather, aggregates grown in high stiffness gels mainly contained hollow, unpolarized cavities. In conditions where polarized lumens did form, we did not find a significant difference in apical actin expression based upon substrate stiffness. Together, these data show that lumen-containing hiPSC aggregates exhibit apical-basal polarity.

### 3.6 Nonadhesive PEG Hydrogels Support hiPSC Pluripotency

**Figure 7:**
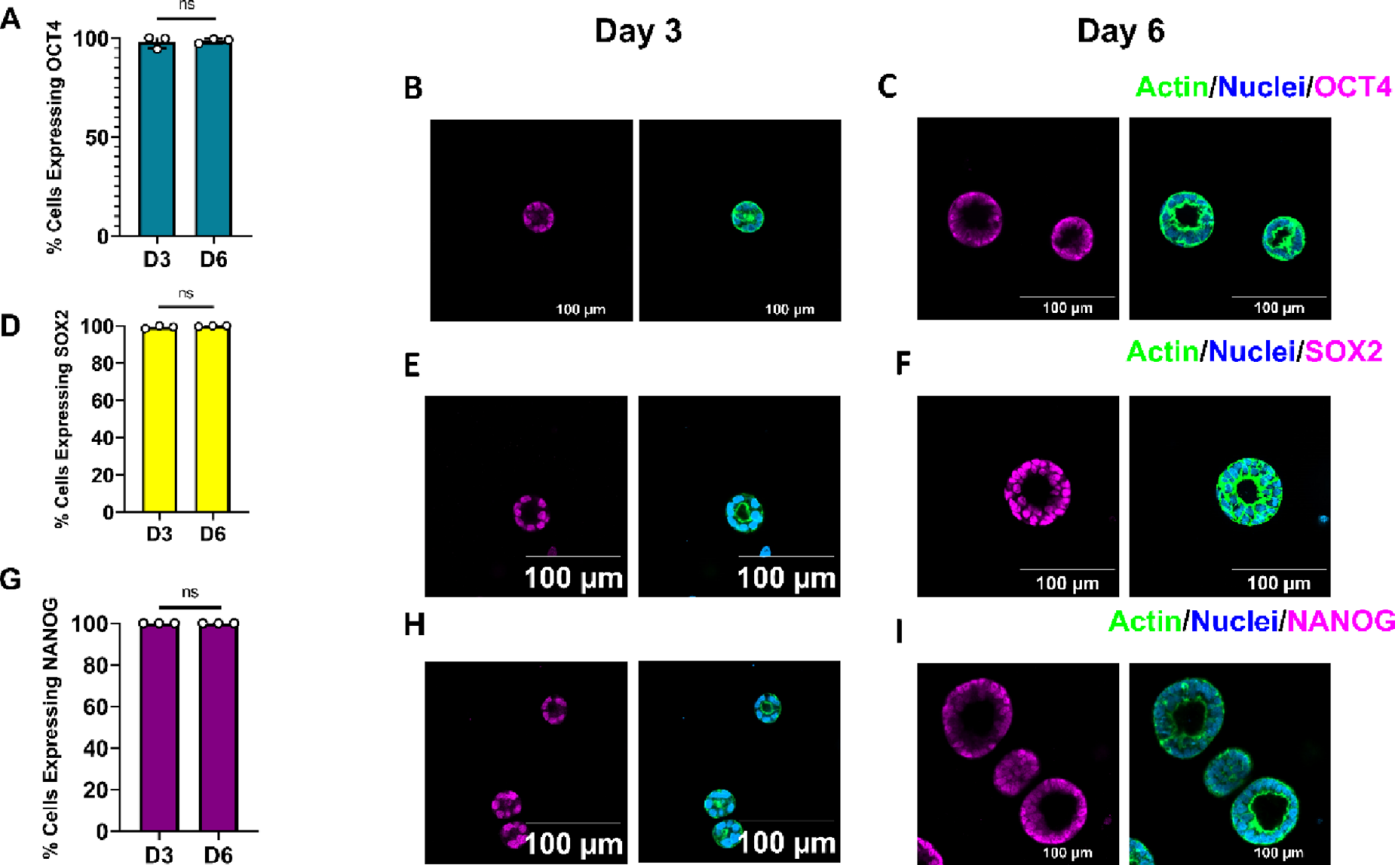
Single hiPSCs encapsulated in PEG hydrogels maintain pluripotency. (A, D, G) Expression of pluripotency factors OCT4, SOX2, and NANOG on days 3 (OCT4 *n* = 525, SOX2 *n* = 233, and NANOG *n* = 152 cells) and 6 (OCT4 *n* = 916, SOX2 *n* = 769, and NANOG *n* = 728 cells) post-encapsulation, represented as the average across each sample (*n* ≥ 3 gels). At least three images were taken per gel. An unpaired, two-tailed *t* test with Welch’s correction was used to analyze the difference in expression between days (ns = not significant, *p* > 0.05). (B, C, E, F, H, I) Representative immunostaining images of each marker. Scale bars are 100 μm.

Given the unprecedented ability of single hiPSCs to form epiblast-like lumen structures in PEG hydrogels devoid of any adherence cues, we wanted to assess the ability of our system to support hiPSC pluripotency. Thus, we analyzed the expressions of OCT4, SOX2, and NANOG via immunofluorescence on days 3 and 6 of culture. Cells were grown in intermediate stiffness gels, as this condition produced the most viable, uniform aggregates with a central lumen. Immunofluorescence revealed that over 99% of cells expressed all pluripotency markers OCT4, SOX2, and NANOG on both days 3 and 6 of culture, with no significant difference being found in expression between time points (Figure 7A–I). This suggests that our minimally instructive matrices can support hiPSC self-renewal in a long-term culture.

### 3.7 PEG Hydrogels Support Directed Differentiation into Three Germ Layers

**Figure 8:**
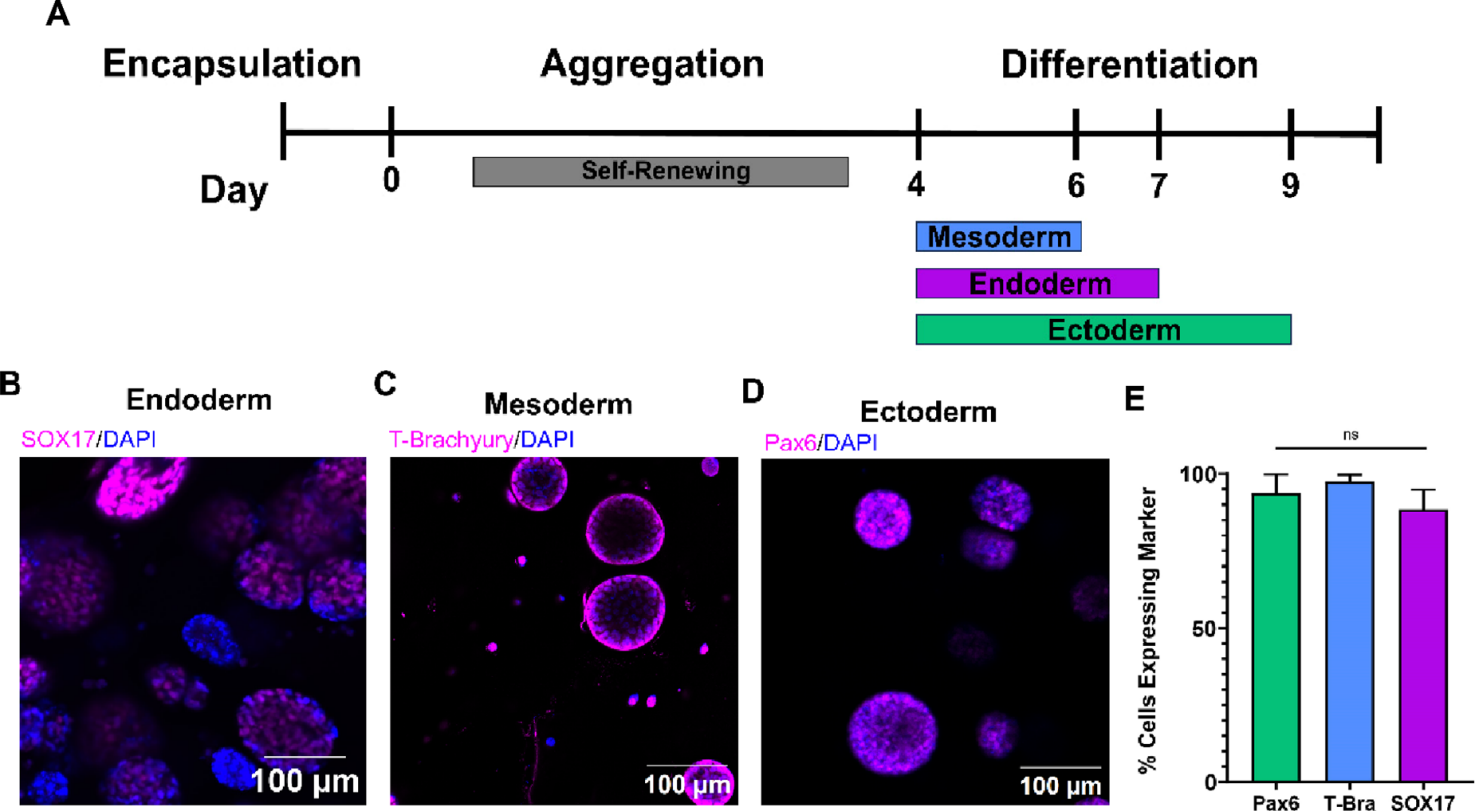
PEG hydrogels were found to support the directed differentiation of the encapsulated RUES2 hESCs. A) Differentiation timelines for endoderm, mesoderm, and ectoderm lineages. (B–D) Representative fluorescent images of aggregates at the end of each differentiation procedure. Scale bars are 100 μm. (E) Expression of germ layer markers PAX6, T-Brachyury, and SOX17 were determined through immunostaining or reporter (SOX17-tdtomato). Expression was represented as the average percentage of expressing cells, averaged across multiple samples (*n* ≥ 3 gels, PAX6 *n* = 1930, T-BRA *n* = 800, and SOX17 *n* = 1889 cells). At least three images were taken per gel. Ordinary one-way ANOVA with Tukey’s post hoc correction was used to analyze for differences in germ layer activation between lineages (ns = not significant, *p* > 0.05).

To assess the differentiation potential of encapsulated cells relative to the epiblast, hPSCs were stimulated with different media and characterized for their ability to differentiate into three germ layers. We utilized a fluorescent germ line reporter (RUES2-GLR) to monitor the differentiation of the cells into ectoderm, mesoderm, and endoderm lineages. Cells were encapsulated at 2.5 × 10^6^ cells/mL in intermediate stiffness gels and grown for 4 days under self-renewing conditions. These cells formed aggregates with a central lumen, similar to the hiPSCs (WTC-11) used in this study, and maintained pluripotency (based on expression of SOX2) upon culture for 4 days (Figure S6). Our findings corroborate earlier studies where RUES2-GLR human embryonic stem cells (hESCs) have been shown to form aggregates with a central lumen in a model epiblast.^36^ After aggregates had formed, E8 medium was replaced with ectoderm (PAX6^+^), mesoderm (T-BRA^+^), and endoderm (SOX17^+^) differentiation media, under constant ROCK inhibition. Cells remained as aggregates throughout the differentiations, with over 85% of cells expressing the respective lineage markers. No significant differences in differentiation efficiency were found between lineages (Figure 8E). To account for differences in the differentiation capacity between hESCs and hiPSCs, this directed trilineage differentiation was assessed with WTC-11 cells. Similar to RUES2-GLR, the WTC-11 cells also showed differentiation into the three germ layers, as indicated by the high percentage expression of respective markers (Figure S7).

## 4 Discussion

Human embryonic epiblast cells self-organize during the early stages of development, forming the lumen of the proamniotic cavity. Despite several hPSC-based models of epiblast morphogenesis, the effect of the mechanical microenvironment on human epiblast morphogenesis remains unclear, primarily due to lack of a tunable biomaterial platform with the ability to independently modulate biochemical and biophysical cues.^9,20,24,25,41,42^ Here, we sought to decouple the biochemical and biophysical matrix cues that influence hPSC morphogenesis by designing defined, nonadhesive PEG hydrogel matrices for iPSC culture. First, we found that “blank-slate” PEG hydrogels could be rendered conducive to iPSC culture by simply tuning their elastic modulus. Encouraged by these results, we next used our matrices as a tool to investigate the role of matrix stiffness in hPSC lumenogenesis. We found that the hydrogel elastic modulus regulates lumen formation, morphology, and apical-basal polarization. Finally, we demonstrated that hydrogel matrices with optimum stiffness could support hPSC pluripotency and trilineage differentiation capacity. Together, these results demonstrate that matrix elasticity is a critical factor in hPSC lumenogenesis and that hPSCs can self-organize in a 3D matrix in the absence of any adhesion cues, thus making nonadhesive PEG hydrogels a robust platform for modeling hPSC morphogenesis and other lumenogenesis events.

To investigate the role of matrix stiffness in iPSC lumenogenesis, we wanted to design a minimal component synthetic 3D culture system that allowed precise control over matrix mechanical properties. Michael-addition cross-linked PEG hydrogels are well-known for their ability to support the culture of many different cell types, including hPSCs, while providing facile tuning of physical properties like stiffness, swelling, and mesh size.^27,43−45^ As such, this chemistry formed the basis of our culture system. Culturing the hPSCs in “blank-slate” PEG hydrogels has some distinct advantages and potential applications for regenerative medicine. First, the gels can be made using a well-defined and tunable one-step chemistry, which is highly cytocompatible. Second, PEG hydrogels present a biologically inert and neutral substrate, thus minimizing effects of other cell–matrix interaction factors such as substrate charges, adhesivity, or similarity to biological molecules. Third, it provides a modular tool to investigate the role of various matrix physical and biochemical properties. While we have not used any adhesion ligands, adhesion peptides of interest can be easily incorporated into PEG hydrogels to further understand the interplay of substrate mechanical stiffness and biochemical factors.^21,22,46^ Lastly, our hydrogels support pluripotency and lumenogenesis in the hPSC culture and may be used to form organoids via directed differentiation after lumens are established. This may be critical in generating organoids with more in vivo-like characteristics, as human embryogenesis occurs after a polarized lumen-containing epiblast is established by the pluripotent stem cells.^9,35^

Although our nonadhesive PEG hydrogels were able to support hPSC viability, aggregation, lumenogenesis, pluripotency, and differentiation in the absence of any matrix biochemical cues, matrix adhesion has been widely regarded as necessary for iPSC survival and proliferation in PEG hydrogels and other engineered hydrogels.^25,30,47,48^ Kloxin et al. investigated the effects of Matrigel inspired adhesion ligands on iPSC viability and differentiation using photo-cross-linked PEG hydrogels, finding β1 integrin binding to promote survival and proliferation.^47^ A later study by Arkenberg et al. showed matrix degradation and adhesion were necessary to support iPSC viability and aggregation in PEG-based hydrogels, as few cells survived in nonadhesive controls.^30^ In this work, we were able to achieve a similar or higher viability without incorporating adhesion ligands. To the best of our knowledge, we are the first ones to show nonadhesive 3D hydrogel matrices support hPSC pluripotency, lumenogenesis, and differentiation.

In our 3D hydrogel matrices, we observed an increase in cell viability with a decrease in hydrogel stiffness (8000–200 Pa; 1.5–5% w/v gels; Figures 2 and S2). As we decreased hydrogel stiffness, a critical viability threshold was reached at a modulus of 1000 Pa, under which stiffness no longer affected viability (Figures 1 and 2). Moreover, we observed that with decreasing hydrogel stiffness, more than 90% of aggregates in soft and stiff hydrogels showed the presence of a single lumen (Figure 3A,B). Similar results were reported by Arkenberg et al., where iPSCs were cultured in gelatin-PEG norbornene hydrogels with a *G*′ of 500 and 1000 Pa.^49^ Cells cultured in the softer matrices had greater viability and an increased prevalence of morphogenic features like lumens. Taken together, this suggests that the threshold we observed may be universal, representing the maximum stiffness that hydrogel matrices can have without impeding hPSC viability and morphogenesis.

Cell–matrix interactions, especially matrix adhesion, have been shown to influence or control lumenogenesis in several different organoid models, including intestinal, kidney, and liver.^29,50,51^ Their role in iPSC lumenogenesis, however, remains unclear. In a seminal work by Taniguchi et al., primed iPSCs cultured in Matrigel were found to recapitulate key features of the human peri-implantation epiblast, forming apical-basal polarized aggregates with central lumens.^9^ As the mechanical and biochemical properties of Matrigel are difficult to control and largely intertwined, defined hydrogel-based iPSC cultures have since been employed to uncover the specific matrix cues that are conducive to lumenogenesis.^24,25^ Here, we show that hPSCs grown in elastic, nonadhesive matrices undergo lumenogenesis and apico-basal polarization, demonstrating that matrix adhesion is not required for these processes to occur in iPSCs. Additionally, we identified the hydrogel elastic modulus as the main matrix cue governing lumen formation and morphology, finding that lumenogenesis and cell polarization only occurred within a narrow range of hydrogel stiffnesses (∼200–1000 Pa) in the absence of any additional matrix cues (Figures 3,4, and 6). While it is difficult to obtain stiffness values for human epiblast, studies in avian embryo indicate that epiblast stiffness ranges from between 100 and 500 Pa, depending on region and development stage.^52^ Thus, the narrow range of optimum matrix stiffness that we observed in our system may be physiologically relevant and is potentially required to support epiblast-like morphogenesis in hPSCs.

While the mechanisms by which nonadhesive 3D hydrogel matrices support hPSC culture and lumenogenesis remain unknown, other adhesion-free cultures might provide some insight.^53,54^ Kim et al. found that iPSCs in suspension were capable of surviving and aggregating through an initial cell–cell attachment phase mediated by E-cadherin, a cell adhesion molecule which regulates iPSC pluripotency and survival.^53,55^ Once assembled, further growth of the aggregate was supported by interactions with secreted collagen I. Therefore, the initial interaction between cells, whether through neighboring cells or clonal growth, may be key to supporting hPSC survival in a nonadhesive system. Consistent with this notion, hPSCs cultured in 3D PEG hydrogels established cell–cell contact, as evident by the E-cadherin staining, regardless of the gel stiffness (Figure 5A). However, those cultured in stiff gels (3% w/v with a modulus of 3000 Pa) failed to form a lumen with high fidelity (Figure 3B) and acquire an apical-basal polarity (Figure 6). This suggests that matrix stiffness not only influences the viability of hPSCs but also governs the rearrangement of cells following aggregate formation. A recent study by Liang et al. identified E-cadherin to be necessary for mouse ESCs to acquire apical-basal polarity and initiate lumen formation both in the absence and presence of ECM cues.^38^ While these results partially explain why cells cultured in soft and intermediate stiffness hydrogels could form luminal aggregates without matrix biochemical cues, they do not account for the vast differences in lumen morphology and cell polarization we observed between hPSC aggregates cultured in soft, intermediate, and stiff hydrogels.

Another critical factor influencing lumenogenesis in epithelial cells is cell division, which is known to guide placement of the apical membrane through orientation of the postmitotic midbody and cytokinetic bridge.^56,57^ Further, a delicate balance between cell proliferation and luminal pressure is shown to regulate maintenance and shape of a lumen in epithelial cells.^58^ In our system, we noted that after formation of a single-layered aggregate with a centrally placed single lumen in the hydrogels with intermediate stiffness, proliferation almost ceased by day 6 (Figure 5E,F). This observation contrasts with the behavior of aggregates in both soft and stiff hydrogels, where proliferation continued (Figure 5E,F). Aggregates in soft gels exhibited smaller, off-centered lumens and multiple layers, while those in stiff hydrogels did not form lumens at all (Figures 3 and 4). The combination of sustained proliferation and the influence of gel mechanical properties on luminal pressure might have played a role in the abnormal morphologies of the lumens observed at these stiffness levels. Additional insights into our findings could be gained by examining research on lumen formation in various epithelial cell types. Studies by Muthuswamy et al. and Debnath et al. showed a notable decline in cell proliferation, as indicated by Ki67 staining, in 3D cultured mammary epithelial cysts upon reaching equilibrium size, occurring on day 6 and day 15, respectively.^59,60^ Petersen et al. found that healthy mammary epithelial cells formed luminal cysts that eventually ceased growing, whereas cancerous cells continued to proliferate, forming solid, nonpolarized spheroids.^61^ Similar results were reported by Patil et al., comparing wild-type MDCK cells and sarcoma virus transfected cells. While the wild-type MDCK cells exhibited controlled proliferation, the sarcoma virus transfected cells underwent increased proliferation, leading to lumen filling.^62^ Lastly, Tanida et al. used computational modeling to explain the origins of different lumen morphologies, such as single-layered and multilayered organoids with single lumens, based on the rate of proliferation and luminal pressure.^63^ Using this model, it was predicted that slow division times and high luminal pressure would result in the formation of single-layered luminal organoids, whereas low luminal pressure or increased proliferation would lead to organoids with multiple layers. Our study suggests that hPSCs cultured in intermediate stiffness gels may have maintained the appropriate balance between cell proliferation and luminal pressure, resulting in the formation of single-layered organoids with a central lumen. Once this process reaches equilibrium, the cell proliferation ceases. However, a delicate balance between cell proliferation and luminal pressure could not be established in hPSCs cultured in soft and stiff gels, leading to the formation of either multilayered organoids with noncentric lumens or unpolarized spheroids lacking lumens. Further support for these hypotheses is derived from the observation that lumen volume scaled more proportionally with the total aggregate volume in intermediate stiffness gels. Conversely, in soft and high stiffness gels, as the aggregates grew larger, the lumen volume did not correspondingly increase (Figure 4C,F). Despite these observations, the specific mechanisms underlying the substrate stiffness-induced differences in cell proliferation remain unclear and warrant further investigation. Nevertheless, our findings highlight the critical role of matrix stiffness in regulating and coordinating both cell proliferation and morphogenetic changes in hPSCs. Although matrix stiffness-regulated differences in lumenogenesis by hPSCs have not been established in prior studies, other lumen forming cell types are shown to exhibit ECM stiffness-regulated lumenogenesis in computational and experiment models. Camacho-Gómez et al. built a model to simulate lumenogenesis, predicting that stiffer matrices would resist the hydrostatic pressure from fluid intake that drives lumen expansion, leading to multilayer aggregates with smaller lumens.^64^ Findings by Enemchukwu et al. also emphasized that lumen formation in MDCK cells is restricted to a narrow range of optimum ECM elasticity, while abnormal morphogenesis is observed at higher or lower elastic moduli.^29^ Our results also corroborate these findings that matrix stiffness is a critical factor regulating lumenogenesis in hPSCs and that hPSCs need an optimal degree of matrix stiffness to form epiblast-like aggregates, under which they organize into multilayered structures with relatively small, nonconcentric lumens, and over which they fail to develop lumens entirely.

Apical-basal polarization is critical for the formation and expansion of physiologically relevant lumens in several cell types, including hPSCs.^9,65,66^ Of the proteins involved in this process, ZO-1 and F-actin have been identified as key mechanosensitive components of lumen formation and expansion, their apical expression regulating morphogenesis at multiple stages of early development.^67,68^ In our study ZO-1 and F-actin staining revealed significant differences in apical expression within aggregates, depending on the stiffness of the hydrogel matrix. Aggregates grown in the stiffest hydrogel matrices did not polarize, rather forming an unpolarized cavity in place of a lumen. Hagelaars et al. demonstrated similar results when culturing MDCK cells on 2D substrates, finding that cells cultured on low adhesion substrates of physiological stiffness (1 kPa) polarized, whereas cells cultured on stiffer substrates (>10 kPa) with high adhesiveness did not.^69^ This is further supported by Enemchukwu et al., who found that matrix degradability increased apico-basal polarization in 3D cultured MDCK cells.^29^ Taken together, in our study, the increased confinement in stiffer hydrogel matrices may have disrupted the hPSCs’ ability to reorganize during proliferation. As ZO-1 has been shown to orient dividing cells, its proper expression is especially important for the formation of a single, central lumen.^39^ The difference we observed in apical ZO-1 and F-actin expression between cells grown in soft and intermediate stiffness hydrogels may be due to a more pliable environment, which allowed greater expression of ZO-1 at the cost of yielding disorganized multilayer aggregates. These findings provide additional support for the notion that matrix stiffness within our system likely dictates the reorganization of cells after aggregate formation.

As the early embryo develops, cells of the epiblast remain pluripotent until the onset of gastrulation.^70^ As such, faithful modeling of the peri-implantation epiblast requires culture matrices that support iPSC pluripotency and controlled differentiation. When hPSCs are 3D cultured in Matrigel, they have been shown to gradually lose their self-renewal capability and demonstrate poor differentiation capacity.^25,32^ Here, hPSCs encapsulated in our minimally instructive hydrogels retain their pluripotency and can robustly differentiate into three germ lines: ectoderm (PAX6 positive), mesoderm (T-Brachyury positive), and endoderm (SOX17 positive cells) only upon induction. Studies in 2D have shown that culturing cells on low adherence substrates upregulates pluripotency.^71^ Indeed, several studies using nonadhesive 3D culture matrices have reported an impressive ability to support hPSC pluripotency.^34,43,72^ While other studies using PEG-based hydrogels have reported similar directed differentiations results, they were performed in the presence of matrix adhesion.^32,47,49^ While outside the scope of this work, the effects of the hydrogel elastic modulus on pluripotency and differentiation may also be investigated using this system, holding promise as a future area of work.

Lastly, both elasticity and viscoelasticity of the matrix are known to influence cell fates and differentiation in various cell types and during embryonic development.^73^ The PEG hydrogels we used in this study are completely elastic, so it is unclear how the viscoelasticity of the substrate in this range of elasticity would impact cell morphogenesis. Our study delves into this intricate relationship, building upon recent findings (Indana et al.) which suggest that viscoelasticity governs lumen formation by hPSCs in alginate hydrogels, while stiffness within the studied range (3–20 kPa) exerts no discernible effect.^25^ However, our results indicated that in a very narrow range, the elasticity of the matrix significantly affects hPSC behavior. Summarizing from our own findings and previous evidence from the literature, we believe if both elasticity and viscoelasticity are varied simultaneously, viscoelasticity may offset the effect of gel elasticity. Thus, our results here offer a novel insight into the range of elasticity that has a significant effect on hPSCs morphogenesis. To study the impact of viscoelasticity and elasticity independent of each other, we will need PEG hydrogels with independent control over viscoelasticity and elasticity. These studies will be critical in determining how the viscoelasticity of PEG hydrogels in this narrow range of optimum stiffness impacts hPSC morphogenesis.

## 5 Conclusion

The nonadhesive PEG hydrogels developed in this study support hPSC viability, self-renewal, lumenogenesis, and trilineage differentiation. While previously thought to be necessary for hPSCs to undergo lumenogenesis, matrix adhesion was not required to form lumenal, polarized aggregates grown from single hiPSCs. Hydrogel matrix stiffness was found to be the chief influencer of lumen formation, morphology, and aggregate polarization, with hydrogels of intermediate stiffness yielding the most physiologically relevant, epiblast-like aggregates. With fine control over hydrogel mechanical properties and the ability to separate biophysical and biochemical matrix cues, this platform presents a powerful tool for understanding how matrix cues influence lumenogenesis in the human epiblast. Since lumen formation is a critical stage in early mammalian development, this simple yet elegant biomaterial platform can provide a powerful model to investigate ECM-regulated developmental processes in a controlled environment. While we demonstrated that adhesion was not required for this process to occur, the effects of matrix adhesion, if any, in this system were outside the scope of this study. As these hydrogels are modular in nature, adhesive ligands could be reincorporated in future studies, thus elucidating the effects of matrix biochemical cues on lumenogenesis in this system.

## Data Availability

The data sets generated and/or used during this study are available from the corresponding author upon request.

## Supporting Information Available

Additional materials and methods; antibodies used in polarization, pluripotency, and germ marker immunostaining; preliminary screening of PEG macromers and thiol cross-linkers; rheological analysis and viability screening of 5% w/v 8-arm PEG acrylate/4-arm PEG thiol hydrogels; effect of ROCK inhibition on hiPSCs cultured in PEG hydrogels; analysis of hPSC aggregate uniformity and circularity in different stiffness PEG hydrogels; schematic of cell alignment and shape analysis; brightfield and fluorescent images of RUES2-GLR hESCs cultured in intermediate stiffness PEG hydrogels under self-renewing conditions; immunofluorescent analysis and quantification of trilineage differentiation capacity in WTC-11 hiPSCs; and fluorescent images of RUES2-GLR and WTC-11 trilineage differentiation isotype controls (PDF)
Supplementary Video 1: animated *z*-stack of aggregates cultured in intermediate stiffness gels, stained for actin (green) and nuclei (blue) (AVI)

## Supplementary Material

ab4c00923_si_001.pdf

ab4c00923_si_002.avi
