# Supplementary material for: Soft Polyethylene Glycol Hydrogels Support Human PSC Pluripotency and Morphogenesis
Source: ACS Biomater Sci Eng. 2024 Jun 19;10(7):4525–40. doi: 10.1021/acsbiomaterials.4c00923 (PMC11234337; doi:10.1021/acsbiomaterials.4c00923)
Supplement: Supplementary file 1 — ab4c00923_si_001.pdf [file ab4c00923_si_001.pdf]

Supplementary Information:

Soft Polyethylene Glycol Hydrogels Support Human PSC Pluripotency and Morphogenesis

Michael P. Seitz,[1,2] Yuanhui Song,[1, 2] Xiaojun Lance Lian,[3] Zhen Ma,[1,2] Era Jain [1,2]

1 Department of Biomedical and Chemical engineering, Syracuse University, Syracuse, NY,

13244, USA

2 Bioinspired Syracuse: Institute for Material and Living System, Syracuse University, Syracuse, NY 13244, USA

3 Department of Biomedical Engineering, The Huck Institutes of the Life Sciences, Department of Biology, Pennsylvania State University, University Park, PA 16802, USA.

***Corresponding Author**
Era Jain (Ph.D.)
Biomedical and Chemical Engineering
Bioinspired Syracuse: Institute for Material and Living System
Syracuse University
Syracuse, NY, USA, 13244
Tel: 315.443.4050
Email: erjain@syr.edu

Supplementary Materials and Methods

4-arm PEG-acrylate (4-arm PEGAc; 10,000 Da), was obtained from Layson Bio. 2-amino butane dithiol (DTBA; 173 Da) was purchased from Sigma Aldrich (St. Louis, MO, US).

Hydrogel Fabrication:

4-arm and 8-arm PEG acrylate containing hydrogels with dithiol crosslinker DTBA were fabricated at a acrylate to thiol molar ratio of 1:1 at 5% w/v total polymer concentration. The procedure for fabricating the hydrogel is the same as described in the main manuscript.

Table S1: Antibodies used in immunofluorescent analysis of pluripotency, polarization, and trilineage differentiation capacity.

Antigen	Isotype	Dilution	Vendor	Product #
OCT4	Rabbit IgG	1:50	Abcam	ab19857
SOX2	Rabbit IgG	1:200	Abcam	ab92494
NANOG	Rabbit IgG	1:50	Abcam	ab21624
T-Brachyury	Goat IgG	1:40	ThermoFisher	PA5-46984
PAX-6	Mouse IgG2a	1:50	Biolegend	862001
ZO-1	Rabbit IgG	1:100	ThermoFisher	402200
E-cadherin	Mouse IgG	1:50	Abcam	ab1416
Ki67	Mouse IgG	1:50	Biolegend	350502
Isotype Control	Rabbit IgG	Assay Dependent	Abcam	ab37415
Isotype Control	Mouse IgG2a	Assay Dependent	ThermoFisher	02-6100

S1. Preliminary screening of PEG macromers and thiol crosslinkers

We screened 4-arm and 8-arm PEG acrylate for their ability to form a hydrogel and support hiPSCs viability post encapsulation. Our results indicated that the PEG hydrogels formed using multi-arm PEG acrylate and dithiol crosslinker (DTBA) showed low cell viability 3 days post encapsulation in the hydrogels. Comparatively hydrogels formed using 8-arm PEG acrylate supported higher cell viability than hydrogels formed using the 4-arm PEG acrylate (**Table S2**). Moreover, we could not form hydrogels of less than 5% w/v polymer concentration using the 4-arm PEG acrylate or 8-arm PEG acrylate when using dithiol crosslinker. Thus, we next screened hydrogels composed of 8-arm PEG acrylate and 4-arm PEG thiol. Unlike hydrogels crosslinked with dithiol crosslinker DTBA, hydrogels crosslinked with 4-arm PEG thiol showed aggregate formation and comparatively higher viability on day 3 post encapsulation (**Figure S2**). Thus, hydrogels formed with 8-arm PEG acrylate and 4-arm PEG thiol were used for further studies.

Table S2: Preliminary screening of PEG hydrogel for their ability to support hiPSCs viability post encapsulation.

PEG Macromer	Thiol crosslinker	Percent gel concentration (w/v)	Storage modulus (G', kPa)	% Cell viability Day 3
**4-arm PEG acrylate**	DTBA[a]	5	0.59 ± 0.003	0.28 ± 0.01
**8-arm PEG acrylate**	DTBA	5	4.6 ± 1.25	3 ± 1.67
**8-arm PEG acrylate**	4-arm PEG thiol	5	2.8 ± 0.742	See S2

Note: **a.** DTBA is a dithiol crosslinkers with two terminal thiols while 4-arm PEG thiol has 4 terminal thiols.

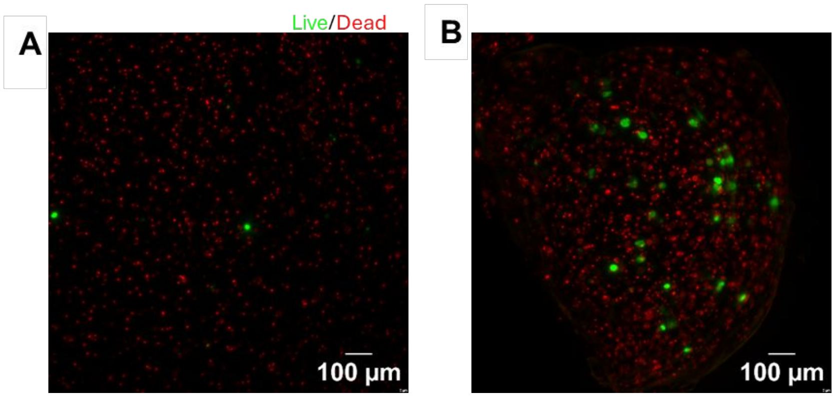

Figure S1: Representative images indicating hiPSCs viability post encapsulation in PEG hydrogels made using DTBA dithiol crosslinker and 4-arm or 8-arm PEG acrylate. Cell viability of hiPSCs encapsulated in PEG hydrogels composed of **A)** 4-arm PEG Ac-DTBA **B)** 8-arm PEG Ac-DTBA. **Single hiPSCs** were encapsulated in 5% w/v PEG hydrogels at a density of $2.5x10^6$ cells/ml and cultured for 3 days. E8 media was supplemented with ROCKi was used. Viability staining was performed, and fluorescent images were captured on a Leica Thunder imager. Scale bars are 100 µm. Cells viability was low in all conditions. While the most viable cells were found in 8-arm gels, no significant difference was found between arms and time points. Ordinary one-way ANOVA with Tukey post-hoc correction was used to analyze each data (ns = not significant; p>0.05).

S2. Rheological analysis and viability screening of 5% w/v 8-arm PEG-Ac / 4-arm PEG thiol hydrogels.

Preliminary screening of 5% w/v 8-arm PEG-Ac crosslinked with 4-arm PEG thiol hydrogels showed that although the encapsulated single hiPSCs can form aggregates, the viability remained low 3 days post encapsulation (**Figure S2C&D**). This may be due to a very high stiffness of these gels compared to gels formed using lower polymer concentration (1.5 – 3% w/v; **Figure 1 and S2A&B**).

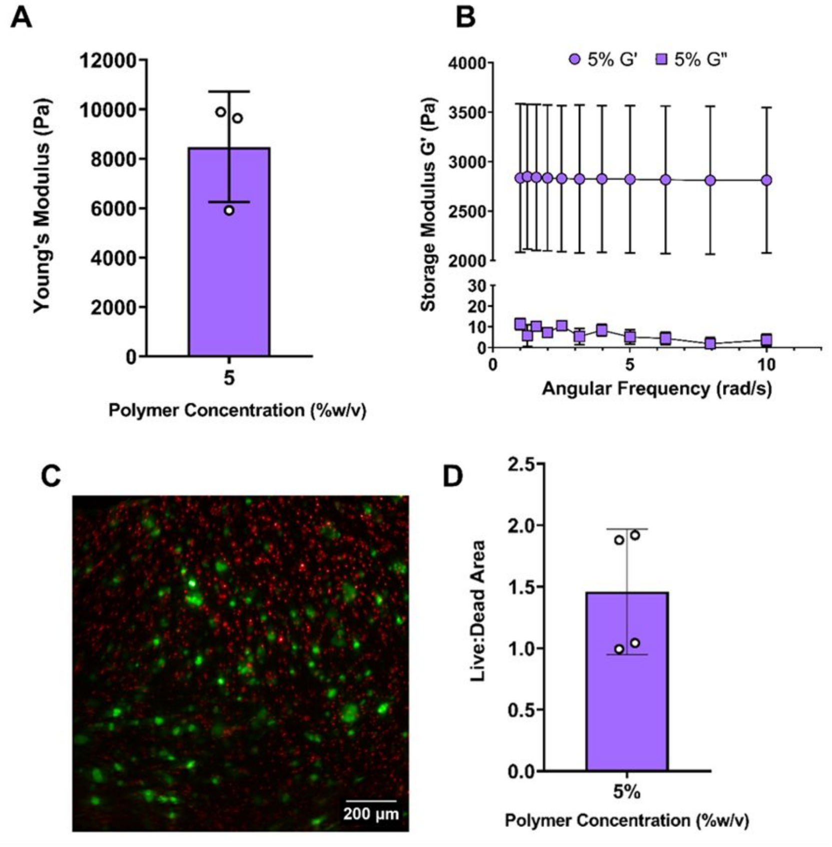

Figure S2: Rheological analysis and viability screening of 5% 8-arm PEG-Ac / 4-arm PEG-SH hydrogels. For all experiments, n = 3 gels. **A)** Young's modulus values were estimated from G' data obtained through rheometery. **B)** A frequency sweep revealed these gels to be mainly elastic. **C)** Single hiPSCs were encapsulated at a density of 2.5x10^6 cells/ml and cultured for 3 days in E8 media supplemented with ROCKi, after which viability staining was performed. Unlike in 5% DTBA gels, aggregates formed after 3 days of culture. Scale bars are 200 µm. **D)** Aggregate viability on day 3 was quantified in ImageJ and presented as the ratio of live to dead cell area.

S3. Effect of ROCK inhibition on hiPSCs cultured in PEG hydrogels

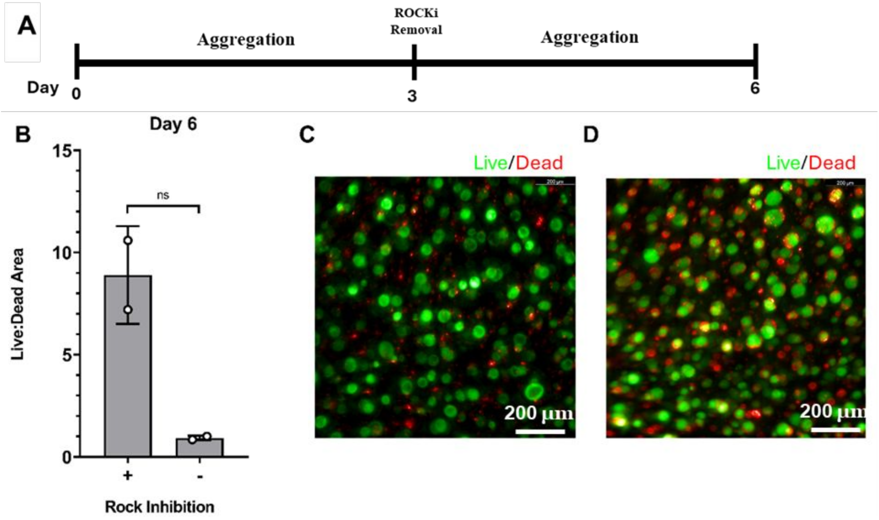

Figure S3: Effects of ROCKi removal on aggregate viability. **A)** Cells were encapsulated in intermediate stiffness gels at a density of 2.5x10^6 cells/ml and cultured under self-renewing conditions with ROCKi. After allowing cells to aggregate for 3 days, ROCKi was removed, and the cells were grown until day 6. **B)** Aggregate viability on day 6 was calculated from fluorescent viability staining images and presented as the ratio of live to dead cell area. N = 2 for all gel samples. Viability data was analyzed using an unpaired, two-tailed t-test with Welch's correction (ns = not significant). **C)** Cells treated with ROCKi until day 6 and **D)** cells grown until day 6 after ROCKi removal on day 3. Aggregates with dead cells were clearly visible with the removal of ROCKi. Scale bars are 200 µm.

S4. Effect of hydrogel stiffness on aggregate size dispersity and circularity

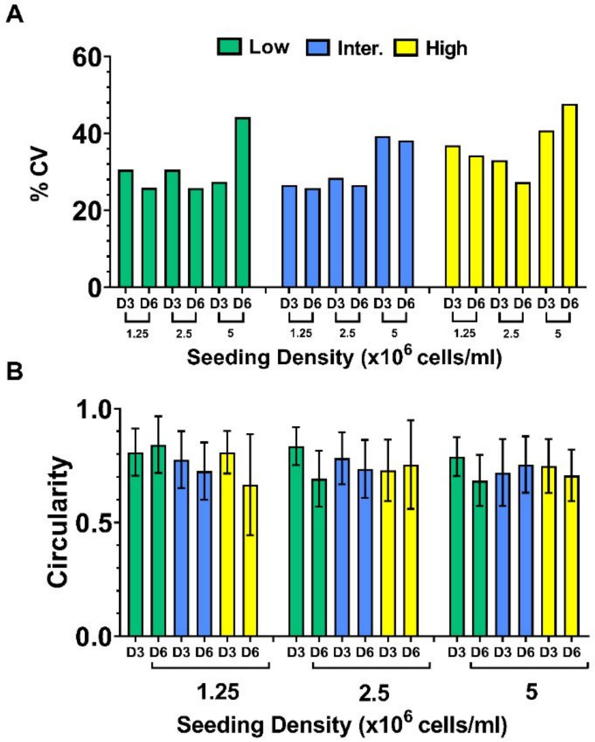

Figure S4: Hydrogel stiffness influences aggregate size dispersity and circularity. **A)** Quantification of polydispersity in aggregate size cell viability from images captured during live dead assay on day 3 and 6 post encapsulation of single hiPSCs. % CV was calculated from aggregate diameters obtained from viability images, using the equation: $\frac{standard\ deviation}{mean} * 100$. **B)** Aggregate circularity was quantified from viability images using Image J. Aggregates were relatively circular in all gels, with no trend being found relative to hydrogel stiffness or seeding density. Viability is represented as the ratio of green to red channel area, averaged across the entire sample (n≥3 gels). Ordinary one-way ANOVA with Tukey post-hoc correction was used to analyze each data (ns = not significant; p>0.05, * p< 0.05, **p< 0.01, and ****p< 0.0001).

S5. Schematic of cell alignment and shape analysis

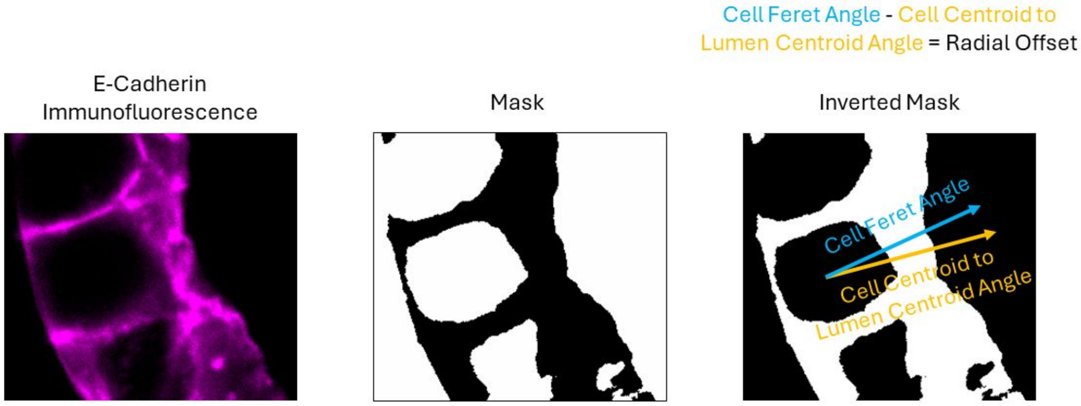

Figure S5: Cell alignment and aspect ratio characterization workflow.

S6. Lumen formation and pluripotency maintenance by reporter cells RUES2-GLR upon culture in PEG hydrogels

Reporter cells RUES2-GLR were encapsulated in PEG hydrogels of intermediate stiffness and culture for 4 days. The cells formed aggregates with a central lumen similar to the WTC-11 hiPSC cell line used in this study (Figure 3A). The cells also maintained pluripotency as indicated by the green fluorescent protein (GFP) reporter for SOX-2 expression. Our finding corroborates earlier studies where the RUES2-GLR were shown to form an epiblast-like lumen in a model of epiblast using a synthetic hydrogel system[34].

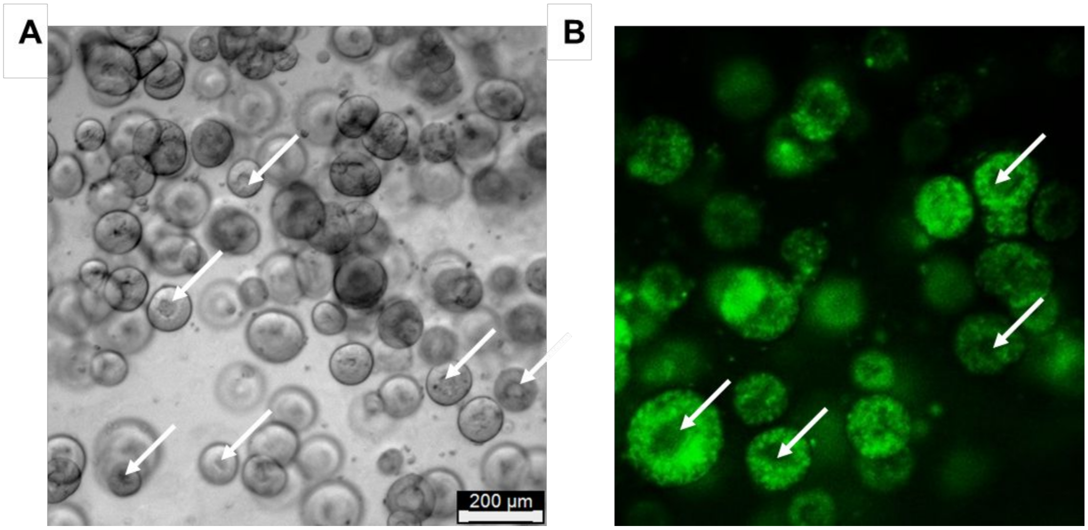

Figure S6: Reporter cells RUES2-GLR show lumen formation and maintain pluripotency after encapsulation in PEG hydrogels of intermediate stiffness. **A)** Representative bright field images showing aggregation and lumen formation in cells cultured for 4 days in PEG hydrogels of intermediate stiffness (2% gels). **B)** Representative fluorescent images showing expression of SOX-2 expression (green) in cells cultured in PEG hydrogels for 4 days. White arrows indicate the presence of lumen in the cell aggregates.

S7. Directed trilineage differentiation of WTC-11 hiPSCs

The trilineage differentiation of WTC-11 hiPSC aggregates was performed using the same procedure as described for RUES-GLR cells. Briefly, cells were first encapsulated at $2.5x10^6$ cells/ml in intermediate stiffness gels and grown for 4 days under self-renewing conditions. Media was then replaced with Stemdiff^{TM} Trilineage Endoderm and Ectoderm media, as the manufacturer's protocol, or CHIR99021 for 48 hours. All germ layer markers were analyzed via immunofluorescence. Similar to RUES-GLR the WTC-11 cells also showed differentiation into the three germ layers as indicated by high percentage expression of respective markers.

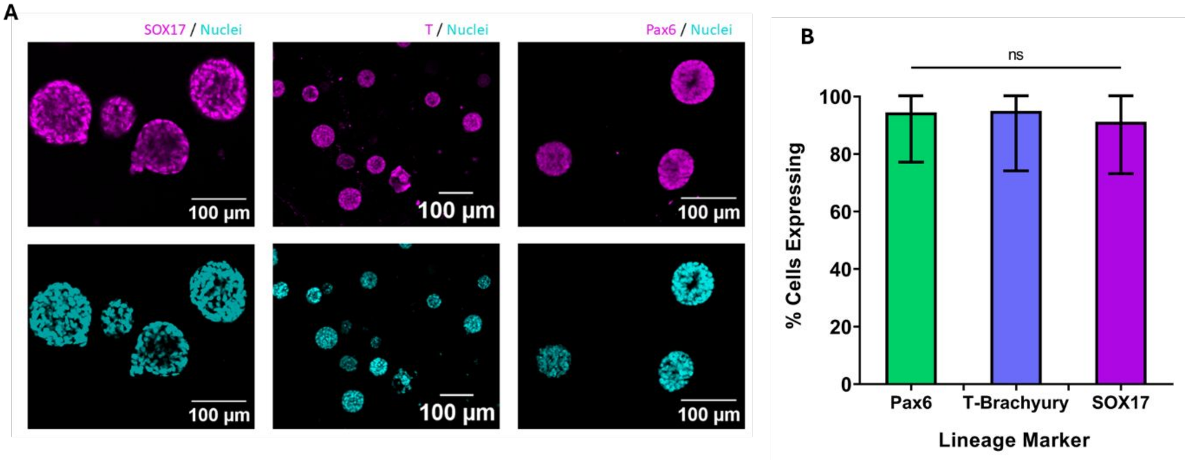

Figure S7: Directed differentiation of WTC-11 hiPSCs encapsulated in intermediate stiffness PEG hydrogels. **A)** Representative fluorescent images of aggregates at the end of each differentiation procedure. **B)** Expression of germ layer markers Pax6, T-brachyury, and SOX17 were derived from immunofluorescence imaging. Expression was represented as the average percentage of lineage marker expressing cells, averaged across multiple samples (n≥3 gels). At least three images were taken per gel. Ordinary one-way ANOVA with Tukey post-hoc correction was used to analyze for differences in germ layer activation between lineages (ns = not significant)

S8. Isotype controls for protein markers used in trilineage differentiation studies

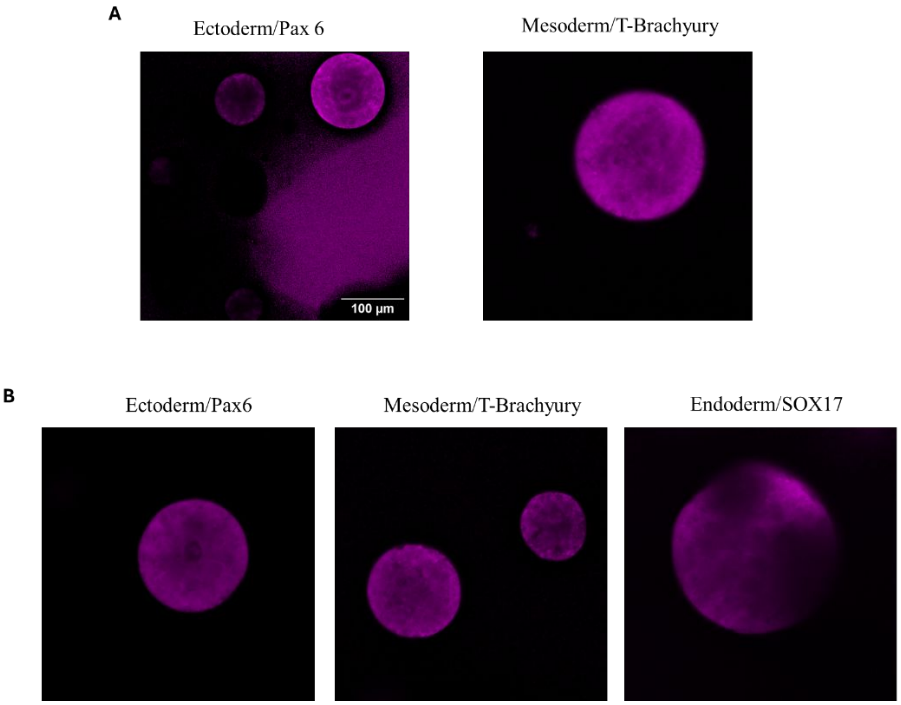

Figure S8. A) Isotype control immunostaining from differentiated RUES-2 GLR cells. **B)** Isotype control immunostaining from differentiated WTC-11 cells.
